# Gene Expansion Shapes Genome Architecture in the Human Pathogen *Lichtheimia corymbifera*: An Evolutionary Genomics Analysis in the Ancient Terrestrial Mucorales (Mucoromycotina)

**DOI:** 10.1371/journal.pgen.1004496

**Published:** 2014-08-14

**Authors:** Volker U. Schwartze, Sascha Winter, Ekaterina Shelest, Marina Marcet-Houben, Fabian Horn, Stefanie Wehner, Jörg Linde, Vito Valiante, Michael Sammeth, Konstantin Riege, Minou Nowrousian, Kerstin Kaerger, Ilse D. Jacobsen, Manja Marz, Axel A. Brakhage, Toni Gabaldón, Sebastian Böcker, Kerstin Voigt

**Affiliations:** 1 University of Jena, Institute of Microbiology, Department of Microbiology and Molecular Biology, Jena, Germany; 2 Leibniz Institute for Natural Product Research and Infection Biology, Department of Molecular and Applied Microbiology, Hans Knöll Institute, Jena, Germany; 3 University of Jena, Department of Bioinformatics, Jena, Germany; 4 Leibniz Institute for Natural Product Research and Infection Biology, Hans Knöll Institute, Systems Biology/Bioinformatics, Jena, Germany; 5 Centre for Genomic Regulation (CRG), Barcelona, Spain; 6 Universitat Pompeu Fabra (UPF), Barcelona, Spain; 7 Centre Nacional d'Anàlisi Genòmica (CNAG), Functional Bioinformatics, Barcelona, Spain; 8 Laboratório Nacional de Computação Científica (LNCC), Petrópolis, Rio de Janeiro, Brazil; 9 Ruhr University Bochum, Department of General and Molecular Botany, Bochum, Germany; 10 Leibniz Institute for Natural Product Research and Infection Biology, Hans Knöll Institute, Department of Microbial Immunology, Jena, Germany; Los Angeles Biomedical Research Institute at Harbor-UCLA Medical Center, United States of America

## Abstract

*Lichtheimia* species are the second most important cause of mucormycosis in Europe. To provide broader insights into the molecular basis of the pathogenicity-associated traits of the basal Mucorales, we report the full genome sequence of *L. corymbifera* and compared it to the genome of *Rhizopus oryzae*, the most common cause of mucormycosis worldwide. The genome assembly encompasses 33.6 MB and 12,379 protein-coding genes. This study reveals four major differences of the *L. corymbifera* genome to *R. oryzae*: (i) the presence of an highly elevated number of gene duplications which are unlike *R. oryzae* not due to whole genome duplication (WGD), (ii) despite the relatively high incidence of introns, alternative splicing (AS) is not frequently observed for the generation of paralogs and in response to stress, (iii) the content of repetitive elements is strikingly low (<5%), (iv) *L. corymbifera* is typically haploid. Novel virulence factors were identified which may be involved in the regulation of the adaptation to iron-limitation, e.g. LCor01340.1 encoding a putative siderophore transporter and LCor00410.1 involved in the siderophore metabolism. Genes encoding the transcription factors LCor08192.1 and LCor01236.1, which are similar to GATA type regulators and to calcineurin regulated CRZ1, respectively, indicating an involvement of the calcineurin pathway in the adaption to iron limitation. Genes encoding MADS-box transcription factors are elevated up to 11 copies compared to the 1–4 copies usually found in other fungi. More findings are: (i) lower content of tRNAs, but unique codons in *L. corymbifera*, (ii) Over 25% of the proteins are apparently specific for *L. corymbifera*. (iii) *L. corymbifera* contains only 2/3 of the proteases (known to be essential virulence factors) in comparision to *R. oryzae*. On the other hand, the number of secreted proteases, however, is roughly twice as high as in *R. oryzae*.

## Author Summary

*Lichtheimia* species are ubiquitous saprophytic fungi, which cause life-threating infections in humans. In contrast to the mucoralean pathogen *R. oryzae*, *Lichtheimia* species belong to the ancient mucoralean lineages. We determined the genome of *L. corymbifera* (formerly *Mycocladus corymbifer* ex *Absidia corymbifera*) and found high dissimilarities between *L. corymbifera* and other sequenced mucoralean fungi in terms of gene families and syntenies. A highly elevated number of gene duplications and expansions was observed, which comprises virulence-associated genes like proteases, transporters and iron uptake genes but also transcription factors and genes involved in signal transduction. In contrast to *R. oryzae*, we did not find evidence for a recent whole genome duplication in *Lichtheimia*. However, gene duplications create functionally diverse paralogs in *L. corymbifera*, which are differentially expressed in virulence-related compared to standard conditions. In addition, new potential virulence factors could be identified which may play a role in the regulation of the adaptation to iron-limitation. The *L. corymbifera* genome and the phylome will advance further research and better understanding of virulence mechanisms of these medically important pathogens at the level of genome architecture and evolution.

## Introduction

The basal lineages of terrestrial fungi, formerly Zygomycota, were recently shown to be polyphyletic and were therefore separated into four separate subphyla [1]. Especially the order Mucorales of the Mucoromycotina encompasses several human pathogenic species. Although infections with mucoralean fungi (mucormycosis) are less common as compared to aspergilloses or candidioses, these fungi are increasingly recognized as the source of infection in immunocompromised patients [2]. Mucormycoses are associated with rapid blood vessel invasion and massive destruction of tissue (necrosis) [3], [4]. Mortality rates are high (∼50%) and treatment mainly includes a combination of antifungals and extensive surgery [2], [5]–[7]. In addition, mucoralean pathogens are resistant to a variety of antifungals including voriconazole which makes treatment even more complicated [8].

The order Mucorales comprises 240 described species, of which at least 20 have been found to be involved in mucormycosis. Genome sequences have been published for only two important pathogenic species within the Mucorales, namely *Rhizopus oryzae* ( = *R. arrhizus*) and *Mucor circinelloides*. These species are closely related and represent derived lineages within the group. However, a large proportion of pathogenic Mucorales (10 species) belong to more basal groups including the genera *Lichtheimia*, *Rhizomucor*, *Apophysomyces*, *Saksenaea* and *Syncephalastrum*. Recently, the first report of the involvement of *Thamnostylum lucknowense*, an ancient mucoralean fungus, in human infections has been published [9]. To date, almost nothing is known about the genomic structure and pathogenicity mechanisms of these basal groups.

*Lichtheimia* species are ubiquitous saprophytic molds and represent the second and third most common cause of mucormycosis in Europe and worldwide, respectively [2], [7], [10], [11]. The genus *Lichtheimia* was formerly included in the genus *Absidia* based on morphological similarities [12]. However, based on the higher growth optimum as well as morphological and molecular data *Lichtheimia* species were separated from the mesophilic *Absidia* species [13]. Today the genus encompasses five thermotolerant species, of which three are known to be clinically relevant, namely *L. corymbifera*, *L. ramosa* and *L. ornata* [14]. In addition to the distinct phylogenetic position at the base of mucoralean fungi, *Lichtheimia* species exhibit differences in physiology compared to the sequenced pathogens *M. circinelloides* and *R. oryzae*, including a higher maximum growth temperature (48–52°C vs 37°C and <45°C) and differences in susceptibility to certain antifungals [8], [15]. Moreover, filamentously growing *Mucor* and *Rhizopus* species have been shown to be able to form yeast cells which were also found in patient material and thus might be of relevance during infection [16]–[18]. In contrast, no yeast-like growth forms of *Lichtheimia* species have been observed to date. In addition, pulmonary *Lichtheimia* infections following solid organ transplantation seem to be associated with a higher risk to develop disseminated disease [19]. Besides its role in human infections, *L. corymbifera* is also believed to be associated with Farmer's lung disease (FLD), a hypersensitivity disorder resulting from frequent contact of mouldy material in agriculture [20]. Nothing comparable has been described for other mucoralean species. In addition to their pathogenicity towards humans, several *Lichtheimia* species are known as contaminants of several food products (e.g. cocoa, peanuts, olive products) [21]–[23]. However, despite the known role of *Lichtheimia* species in infection and diseases, several *Lichtheimia* species play an important role in the fermentation of soy products in Asian cuisine [24].The large evolutionary distance and notable differences in infection strategies between *Lichtheimia* and the two sequenced mucoralean pathogens indicate that they independently evolved their ability to infect humans by developing specific pathogenesis mechanisms. To gain insight into the genomic differences between these groups of pathogens, here we report the genome sequence of the type-strain of *L. corymbifera* (FSU 9682, CBS 429.75, ATCC 46771) which has been shown to be a typical strain in terms of virulence and physiology for this species [25] and compare it to published genomes of mucoralean fungi and other fungal phyla.

## Results/Discussion

### Genome assembly and structure

The genome of the type-strain of *L. corymbifera* (FSU 9682, CBS 429.75, ATCC 46771) was sequenced by a combination of 454 sequencing of a shotgun and 8 kb paired-end library in combination with Illumina sequencing of a paired-end read library (Materials and methods, Table S1). The final assembly comprises 209 scaffolds with a N50 scaffold size of 367,562 nt and a total length of 33.6 Mb (Table 1), which is comparable to the genome size of other zygomycetous fungi [26]. Mucoralean genomes are generally believed to contain large amounts of repetitive elements representing around 35% of the genome [27]. However, analysis of the *L. corymbifera* genome shows a much smaller content of repetitive elements, with only 4.7% of the assembly representing repetitive elements including DNA transposons, LTR and non-LTR retrotransposons (Table S2). This finding is consistent with the results of the k-mer analyses on the Illumina reads where only low amounts of potential repetitive regions were found. Of note, all previous estimates of repetitive elements in mucoraleans correspond to species with large genomes such as *R. oryzae* (46 Mb; 20% repetitive elements), *Absidia glauca* (52 Mb; 35%) and *P. blakesleeanus* (54 Mb; 35% repetitive elements) [27], [28]. Interestingly, a *Lichtheimia*-specific gene expansion in the heterokaryon incompatibility genes was discovered (see section gene expansion) which are involved in the recognition of non-self DNA and may contribute to the low amount of repetitive elements. Another mechanism of protection against transposons and viruses is RNA interference resulting in sequence specific RNA degradation [29]. Several predicted proteins with functional domains associated with this mechanism were found including a dicer-like protein, one argonaute-2 protein and a translation initiation factor 2C homolog. However, the exact effects of these mechanisms on the amount of repetitive elements remain to be determined.

**Table 1:** Statistics of the *L. corymbifera* genome.

**Assembly statistics**	
Total scaffold length (Mb)	33.6
Scaffolds	209
N50 contig length (nt)	66,718
N50 scaffold length (nt)	367,562
G+C content	43.4%
**Predicted protein-coding genes**	
Predicted genes	12,379
Average coding sequence size (nt)	1,287
Average G+C content in coding sequence	46.2%
Total introns	48,663
Introns per gene (median)	4.8
Average intron length (nt)	258
**Predicted non-coding RNA genes**	
Predicted genes	213
Average G+C content in non-coding RNAs	49.2%
Total introns	3

Heterozygosity was shown for several fungi including the basal lineage fungus *Batrachochytrium dendrobatidis* [30]. In order to test for potential heterozygous regions and estimate the genome size of *L. corymbifera*, k-mer analyses based on the Illumina reads were performed using an algorithm described previously [31] (Material and Methods). Analysis resulted in a relatively clear single peak with a slightly trailing left flank for all k-values (Figure S1). The distribution could be dissected into three components, each showing a normal distribution with similar variance, but different means and different proportions. The main component represents the potential homozygous part of the genome (94%), whereas two small components represent the potential heterozygous part of the genome (4%), and most likely some repeat regions that occur at relatively low frequency (2%). It has to be noted that the potential heterozygous part is rather small and could as well be explained e.g. by regions that are difficult to sequence and therefore have lower k-mer coverage. The lack of heterozygocity is in accordance with the general assumption that mucoralean fungi are haploid during vegetative growth. Based on the k-mer analysis for different k-mer lengths (41, 59, 69, and 79 nt) a total genome size of around 35 Mb was predicted which is close to our total scaffold length of 33.6 Mb (96% of k-mer predicted size).

### Non-coding RNA prediction and annotation

We annotated 174 tRNAs in *L. corymbifera*. Although *R. oryzae* (239) comprises many more tRNAs, we found unique anticodons among the basal fungi in *Lichtheimia*: CCC (Gly), AAA (Phe) and GAT (Ile). In contrast, only *L. corymbifera* misses the anticodons CAC (Val), CCT (Arg) and TAT (Ile). Three GTA (Tyr) tRNAs were predicted with introns in *L. corymbifera*, while the number was higher in other mucoralean fungi (up to 10). No selenocysteine and possible suppressor tRNAs were predicted. We found the downstream half of 28S rRNA only, but no 18S rRNA in the current assembly. We expect at least two operons (18S – 5.8S – 28S rRNA) as found in *R. oryzae*. In addition to 5S rRNAs located close to the operons, we were able to identify several independent 5S rRNA copies (Table S3). Another housekeeping ncRNA, present in all kingdoms of life, is the ribozyme RNase P, which processes tRNAs by cleaving off nucleotides on the 3′ end of tRNAs [32]. We detected this gene as expected in a single copy per genome, but two identical copies are apparently present in the genome of *R. oryzae*, which may result from whole genome duplication in *R. oryzae* [28]. The pseudoknot in the centre of the molecule is accredited with the catalytic function and highly conserved in evolution [33]. However, the *L. corymbifera* candidate varies exceptionally in sequence, while the secondary structure is maintained. Whether the function of the molecule is affected has to be analyzed. The evolutionary related RNase MRP was invented at the origin of eukaryots with dual function: (a) initiation of mitochondrial replication and (b) separation of 18S rRNA from 5.8S rRNA [34]. One copy per basal fungal genome was detected. The signal recognition particle containing a ncRNA part (SRP RNA) guides proteins to the endoplasmatic reticulum [35]. One copy was detected in *Lichtheimia*, whereas two copies were identified in the genome of *R. oryzae*. Surprisingly, the covariance model of mucoralean fungi, in agreement with *Rhizopus*, *Batrachochytrium* and *Monosiga* (RF00017) is much closer related to metazoans than to other known fungi SRP RNAs. We detected the RNA components of the major spliceosome and collected indications for a functional minor spliceosome. Except for U4 snRNA all five RNAs involved in U2-splicing were detected in *Lichtheimia*. U4 snRNA was not part of the assembly; however an U4-candidate was identified in the originally sequenced read data. Additionally, four of five RNAs involved in AT-AC-splicing were found. However, several special secondary structures were discovered, which may alter the functionality of the minor spliceosome: (i) The third stem of U12 snRNA is atrophied and the last stem is shorter than expected for all basal fungi. (ii) U4atac is not detected in *Lichtheimia*. The other basal fungi show one inconspicuous copy, which is assumed to to be an assembly mistake. However, no similar homologous gene was detected in reads either. (iii) The second half of U6atac is highly divergent (Figure S2 and supplemental material: http://www.rna.uni-jena.de/supplements/lichtheimia/index.html). Eleven CD-box snoRNAs and 3 H/ACA snoRNAs were identified, which are mainly conserved in sequence and structure among basal fungi. For further details we refer to the supplemental material (www.rna.uni-jena.de/supplements/lichtheimia/index.html). Additionally, several ncRNA candidates could be proposed, which have to be functionally characterized in future experiments. A riboswitch, binding to thiamine pyrophosphate (TPP) was found in all basal fungi. For *Lichtheimia* a potential telomerase RNA is suggested, which is surprisingly closely related to the shortest known telomerase RNAs in ciliates (150 nt *Tetrahymena paravorax*). This is unexpected, since the longest telomerase is known from the fungus *Saccharomyces cerevisiae* (1,220 nt). Although the alignment of the usually extremely divergent telomerase RNA is very convincing in sequence and secondary structure (see supplemental material), no homologs in another basal fungus and no interacting ciliate protein homolog were found in our current assembly. U7 snRNA is known to interact with the downstream region of histone mRNA for inhibition of degradation. Four similar candidates for this short ncRNA were identified. In eukaryotes, polymerase III transcripts (e.g. U6 snRNA, RNase P, RNase MRP, SRP RNA, U6atac snRNA) usually display a typical promoter region: −10 nt TATA box, PSE element, Oct region [36]. Therefore, a search for conserved motifs was conducted in *Lichtheimia* promoter regions. However, we were not able to identify even one of these motifs. This highlights a possible modified polymerase III activity for basal fungi and has to be investigated in detail in further work. A phylogeny among basal fungi and *Schizosaccharomyces pombe* as outgroup based on ncRNAs (except 18S and 28S rRNA) was reconstructed, see Figure S2 (B and C). In accordance with protein and traditional rRNA phylogeny *Lichtheimia* groups basal to *P. blakesleeanus* and the other two investigated fungi.

### Protein-coding gene prediction and annotation

To aid prediction of protein-coding genes, RNA-seq analyses were performed for three different growth conditions in three biological replicates (see Material and Methods). The use of different conditions should ensure a higher number of expressed genes, thereby allowing evidence-based gene predictions for many gene models. On average, each replicate has a 70-fold genome coverage, which sums up to a 630-fold genome coverage (Table S4). Prediction of protein-coding genes was performed using AUGUSTUS [37], resulting in 12,379 predicted genes. Genes were functionally annotated by comparing to GenBank sequences using BLASTp (E-value≤10^−25^), and by scanning for the presence of conserved domains using the InterProScan function of BLAST2GO [38]. BLAST hits were obtained for 7,917 genes, InterProScan results were found in 10,066 genes and at least one Gene Ontology (GO) term was assigned to 7,435 genes based on the union of BLAST and InterProScan results. The raw reads of the DNA- and RNA-seq experiments, the final genome assembly, the structural and functional gene prediction are available at http://www.ebi.ac.uk/ena/data/view/PRJEB3978). The genome data are also accessible *via* HKI Genome Resource (http://www.genome-resource.de/).

### Comparison of protein-coding genes between *L. corymbifera* and other completely sequenced genomes

**Figure 1:**
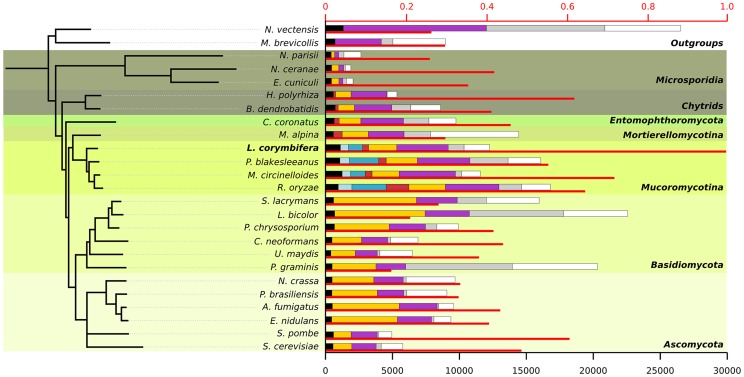
Species tree including the 25 species used during phylome reconstruction. For each species the thin red bar represents the proportion of proteins that have a homolog in *L. corymbifera* (upper axis). The coloured bars represent the number of proteins found in different ranges of species (lower axis): black: wide-spread proteins found in at least 23 of the 25 species, light blue: proteins found exclusively in all four Mucorales species, darker blue: proteins found only in Mucorales species, red: proteins found in early diverging fungi, yellow: proteins found in fungi, purple: proteins found in fungi and at least one of the outgroups, grey: species-specific proteins without orthologs in other species but with paralogs within the genome, white: proteins with no homologs. All nodes in the tree have a bootstrap support of 100.

An exhaustive comparison of *L. corymbifera* genome with other 24 completely sequenced genomes including the major fungal groups (Chytridiomycota, Mucoromycotina, Asco- and Basidiomycota) was performed. This comparison included the reconstruction of *L. corymbifera* phylome, which encompasses the complete set of evolutionary histories of *L. corymbifera* genes (Material and Methods). It was carried out using the previously described PhylomeDB pipeline [39]. In brief, for each *L. corymbifera* protein-coding gene we searched for homologs, and multiple sequence alignments were built, and Maximum Likelihood analyses were performed to reconstruct a phylogenetic tree. The phylome is available through phylomeDB (http://phylomedb.org), with the phylome ID 245. The phylome was used to establish phylogeny-based orthology and paralogy relationships among genes in the species considered [40], and to detect gene expansions (see below). In addition, we used two complementary approaches, gene concatenation and super-tree [41], to reconstruct the species tree that represents the evolution of the 25 species considered. In the first approach, 58 genes were selected that were present in 21 out of 25 in single copy. Their corresponding alignments were then concatenated and a maximum-likelihood species tree was reconstructed (Material and Methods). In the second approach, 9,478 trees present in the phylome were used to build a super-tree using a gene tree parsimony approach, a method which finds the topology that minimizes the total number of duplications in the phylome [42]. Both resulting trees presented a similar topology, which placed *L. corymbifera* at the base of the other Mucorales species (Figure 1). The only difference found between the trees by the complementary approaches was the position of *Schizosaccharomyces pombe*, which appeared at the base of Ascomycota in the super-tree tree while in the concatenated tree it grouped with *S. cerevisiae*. To assess the level of overlap in genetic content between the different species an all-against-all comparison of the 25 genomes was performed. The results indicate between 50% and 75% of the proteins encoded in the other three Mucorales species had homologs in *L. corymbifera* (Figure 1). Surprisingly, this percentage of shared gene content with *L. corymbifera* was similar to that of *Schizosaccharomyces pombe* (60.6%), which is higher than that found in the more closely related chytrid *B dendrobatidis* (an average of 41.1%). Figure 1 also shows how these homologs are distributed in differently defined groups. Most interestingly, the fraction of species-specific proteins (grey bars in the figure) is particularly high in large genomes (e.g., over half of the largest genomes *Laccaria bicolor* and *Puccinia graminis*). Over 25% of the proteins apparently are specific for *L. corymbifera*.

### Conserved gene regions in *L. corymbifera* and other mucoralean genomes

**Figure 2:**
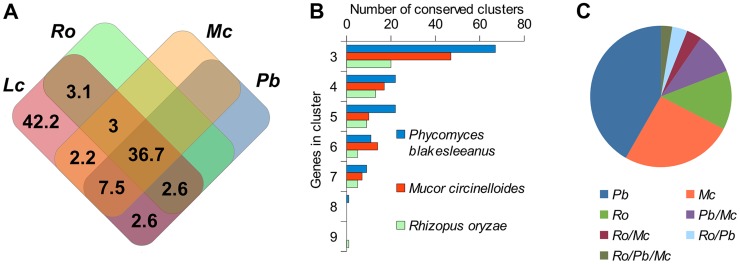
Conserved regions of the *L. corymbifera* genome with other mucoralean genomes. (A) Venn diagram of shared gene families between *L. corymbifera* and other mucoralean fungi based on GhostFam gene families. Numbers indicate percentage of *L. corymbifera* gene families. (B) Number and size of conserved clusters of *L. corymbifera* with other mucoralean genomes. (C) Proportions of conserved clusters of *L. corymbifera* shared with different mucoralean genomes. Occurrences in more than one of the genomes are indicated by a slash between the species.

Since the *Lichtheimia* lineage separated early in mucoralean evolution we can expect that severe genomic re-arrangements have taken place during evolution, causing substantial differences between the genome structures of *L. corymbifera* and other Mucorales. Only 57.7% of the gene families present in *Lichtheimia* are also present in at least one of the other mucoralean genomes while only 36.7% were found in all four genomes representing 70.4% and 53.7% of the total *L. corymbifera* genes, respectively (Figure 2 A). Conserved regions, in terms of gene order, between mucoralean genomes were examined and evaluated with respect to the amount of conserved genes of these regions. A total of 230 regions with a minimum of 3 conserved genes of *L. corymbifera* were found that were present in at least one of the other genomes. These regions were interspersed over 41.1% of the scaffolds but covered only 7.6% of the *L. corymbifera* genome reflecting the high dissimilarity between the mucoralean genomes (Figure 2 B). Only 6 regions were shared with all species. The total number of shared clusters was found to be consistent with the phylogenetic distance between the species (Figure 2 B+C). Genes in the conserved regions are members of different gene families and contain a variety of functional domains.

### Gene duplications in *L. corymbifera*

**Figure 3:**
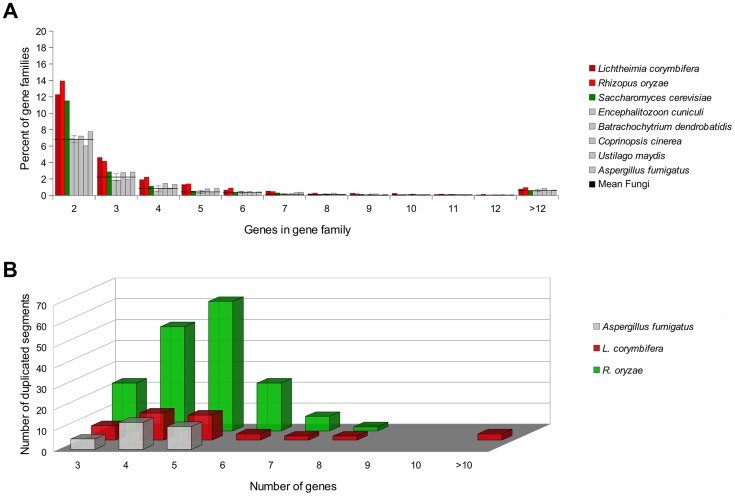
Gene duplication and duplication of genomic regions within mucoralean genomes in comparison to the genome of *A. fumigatus*, which (i) inhabits the same natural habitats and (ii) causes similar symptomatology in human like *L. corymbifera* and (iii) serves as model organism for causative agents of invasive mycoses [56], [75]. The genome of *A. fumigatus* serves as measure for low incidences of singular and segmental gene duplications [115], [116]. (A) Comparison of gene families between *L. corymbifera*, *R. oryzae* and non-mucoralean fungi. Gene families are based on GhostFam homology families. Values for *L. corymbifera* and *R. oryzae* are excluded from the “mean fungi” value. (B) Regions with a minimum of 3 genes were tested for multiple occurrences within the genomes by GECKO2.

In accordance with former results [28] a higher number of gene families with two members were detected for *R. oryzae* but also for *L. corymbifera* as compared to other fungi (6.99%±0.58%) (Figure 3 A). Whole genome duplication has been previously described for *R. oryzae* based on the presence of gene duplications and duplication of large genomic regions (segmental duplications) [28]. To investigate whether segmental duplications and thus a potential WGD also occur in *L. corymbifera*, the genomes were scanned for the presence of duplicated regions using GECKO2 [43], [44]. Consistent with the former findings, our analysis showed a high number of segmental duplications in *R. oryzae* covering more than 10% of the genome [28] while fewer duplicated regions were found in *L. corymbifera* covering less than 4% of its genome (Figure 3 B). Thus, the gene duplications seem not to result from recent WGD as in *R. oryzae* but may result from an ancient genome duplication in mucoralean fungi as suggested by Marcet-Houben et al. [45] which is no longer detectable in the duplicated gene clusters. The possibility of ancient WGD in mucoralean genomes is currently investigated in more detail (Corrochano et al., pers. comm.). The genome of *L. corymbifera* also shows increased numbers of gene families with a higher number of genes indicating that gene duplication and the preservation of the gene copies seem to be a common process in mucoralean genomes and may be independent from WGD. This will be further addressed in the next section.

### Gene expansions in the *L. corymbifera* genome

**Figure 4:**
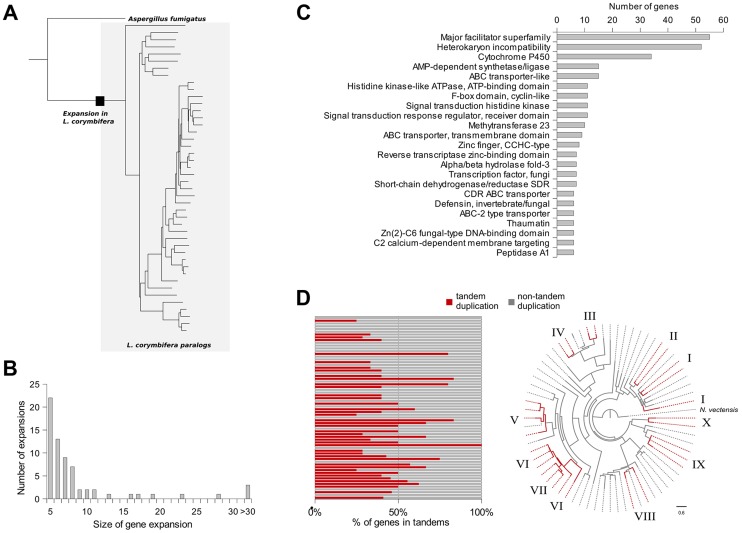
Gene expansions and tandem duplications found in *L. corymbifera*. (A) Tree representing an expansion of HET proteins in *L. corymbifera*. Branches enclosed in the grey shaded area represent paralogs of *L. corymbifera*. The black square represents the point where the expansion started. (B) Number and size of gene expansions in the *L. corymbifera* genome. (C) Main functional domains of gene expansions based on PFAM annotation. The numbers of genes with the different functional domains were combined if a domain was present in more than one expansion. (D) Proportion of genes within gene expansions which are arranged in tandem duplications. Each bar represents an expansion with the red part as the percentage of tandem duplicated genes (left). Clustering of tandem duplicated genes of cytochrome P450 genes in *L. corymbifera* (based on reconstruction with RaxML [130]). Red branches represent tandem duplicated genes. Numbers at the branch tips indicate different tandems.

In addition to gene duplications shared by all mucoralean fungi a high amount of species-specific duplications was detected. Therefore, the phylome was scanned in search of expansions of protein families that occurred specifically in *L. corymbifera*. For each tree, ETE [46] was used to find nodes that contained at least five *L. corymbifera* sequences and no other fungal sequence (Figure 4 A and B). Overlapping expansions were fused when they shared more than half of their members. We found 75 expansions that fulfilled those requirements. Five expansions contained more than 30 members, with the largest containing 331 paralogous genes. In contrast, the large genome of *R. oryzae* contains approximately twice the number of expansions, with the largest encompassing 1,888 members. As some of those expansions are likely the result of the presence of transposons, we scanned them for the presence of transposon-linked domains using the Pfam database and HMMER3 [47], [48]. If the expansions that contain transposons were excluded, 66 groups of paralogous proteins will be left in *L. corymbifera* comprising a total of 820 genes (Figure 4 B).

The most abundant expanded groups (with 331 and 242 members) are rather heterogeneous in terms of functional domains, thus there is no particular function that could be assigned to them. The largest group with a dominating domain contains in total 56 members, of which 52 possess a heterokaryon incompatibility protein (HET) domain (PF06985) (Figure 4 C). Interestingly, this domain was so far attributed nearly exclusively to ascomycetes (with only one exception for the basidiomycete *Moniliophthora perniciosa*), where the HET proteins control somatic allorecognition (non-self-recognition) during the formation of heterokaryons [49]. However, Mucorales, opposite to ascomycetes, do not form heterokaryons by fusion of somatic cells but only during sexual reproduction and zygospore-formation. Since HET domain proteins are absent in all other sequenced zygomycetous genomes it is unlikely that they play a general role in the sexual reproduction but seem to be specific for *Lichtheimia*. Interestingly, these HET genes were differentially regulated under stress conditions. Several copies of the HET domain proteins were down-regulated under iron-depletion and hypoxia. Since these genes are absent in all other mucoralean fungi it is unclear which functions they serve in *L. corymbifera*. In addition it is unclear where these genes originate since they do not occur in other basal fungi and show only very weak similarity with the HET proteins of dikaryan fungi.

Several expansions contain transporters: major facilitator superfamily (MFS, PF07690, PF12832, PF05977, PF13347), ABC transporters (PF00005, PF00501, PF01061, PF00664, PF06422), sugar (and other) transporters (PF00083). In addition, some interesting expansions are connected to the transcription regulation function, which is discussed in more detail in a separate section and signal transduction pathways (see supplemental Material, Table S5 and S6). Four expanded groups are characterized by the cytochrome P450 (PF00067) domain (Figure 4 C). Interestingly, mucoralean pathogens like *L. corymbifera* have been shown to be resistant to several antifungals including voriconazole [8], [50] which could be explained by high copy numbers and isoforms of the target genes. Thus, gene duplication and expansion might be important for the success of *L. corymbifera* in human infections.

Strikingly, these domains (MFS transporters, HET and cytochrome P450) were also the dominant domains in genes which were localized in tandem duplications (Figure S3). Tandem duplicated genes were found to be present in 42 of the 66 gene expansion groups covering 38% of all genes in the expanded groups (Figure 4 D). However, additional smaller tandems were found which did not fit the criteria of gene expansions. A total of 701 genes are organized in such tandem repeats (Figure S3). In addition, duplicated genes were frequently found to be located on the same scaffold which may result from older tandem duplications.

Thus, tandem duplications and a high amount of gene retentions may give an additional explanation for the high amounts of duplicated genes in *L. corymbifera* comparable to the observations in plant genomes where segmental duplications (resulting from WGD) and tandem duplications play different roles in the enrichment of genes of several gene families [51], [52]. Tandem duplication and the retention of duplicated genes would be an explanation for the severe differences in the size of gene families between mucoralean fungi with only 53% of gene families with the same size in *L. corymbifera* and *R. oryzae* (see Figure S3 C).

To investigate if the different gene copies may have different functions and thus may contribute to rapid adaptations to different environmental conditions we analysed the expression of tandem duplicated genes under infection-associated stress conditions (iron depletion and hypoxia; see Table S7). Differential expression of at least one gene of the tandem clusters under at least one of the conditions was found for 71 tandems. Strikingly, only 7 tandems were co-regulated while in 64 cases expression of the copies was different including six cases were copies were antithetically regulated (Figure S4). These results are consistent with the hypothesis that the high prevalence and maintenance of duplicated genes leads to diversification of gene functions.

### Alternative splicing

Duplicated genes can lead to the diversification of gene functions of the two copies which has been discussed in the section above. In addition, alternative splicing (AS) can increase the functional diversity. Gene prediction resulted in 841 alternative splicing events in a total of 683 genes (5.5% of total genes) comparable to the situation in *S. cerevisiae* [53]. Based on the analysis of the RNAseq data alternative splicing could be verified for 273 genes (2.2% of total genes) (Figure S5 A and Table S8). Alternative donor and acceptor are the dominant groups of alternative splicing events (>75% of the total events) which is similar to the situation in several higher eukaryotes [54] (Figure S5 A). Comparison of alternatively spliced genes with genes in tandem duplications and gene expansions showed that only 12 (4.4% of genes with AS) in these groups are also alternatively spliced. If AS occurs in tandem duplicated genes, it occurs in only one of the copies except in one case. This is in accordance with recent results in *S. cerevisiae* which show that duplicated genes can replace one alternatively spliced gene and that alternative splicing is often lost after gene duplication [55]. To test if AS plays a role in the stress adaptation of *L. corymbifera* we analysed the potential alteration in alternative splicing pattern during stress adaptation. Significant changes were only detected for 16 and 23 genes under iron depletion and hypoxia (<0.2% of the total genes), respectively (Figure S5 B). Based on the high incidence of gene duplication and the differential expression of the copies as well as the comparably low number of alternatively spliced genes, maintenance of duplicated genes seems to play a more important role for the generation of functionally distinct paralogs than alternative splicing.

### Identification and expression of potential virulence factors under infection-related conditions

#### a) Iron uptake genes

**Figure 5:**
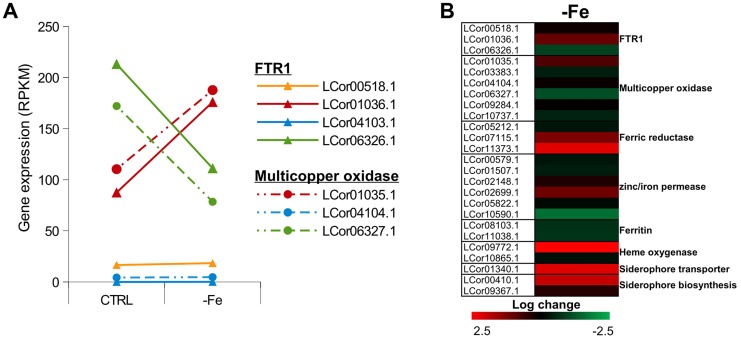
Expression of iron uptake genes under iron limited conditions. (A) Expression levels of FTR1 domain genes and their corresponding multicopper oxidases under standard conditions and iron limitation. (B) Heat map showing the regulation of iron uptake genes under iron-limited conditions.

Iron is an essential trace element for all organisms and plays a crucial role in fungal pathogenicity [56]–[58]. Elevated host iron levels are an important prerequisite for mucormycosis and the iron permease FTR1 has been shown to be crucial for virulence in *R. oryzae* [59]–[62]. The genome of *L. corymbifera* contains four copies of FTR1, of which three are located next to a multicopper oxidase and may share the same promoter. Both FTR1 and multicopper oxidase are important players of the reductive pathway and have been shown to form functional complexes in *C. albicans* [63]. Thus, co-expression of both genes and maintenance of their proximity may contribute to effective iron uptake and, hence, to the virulence of *L. corymbifera*. The higher copy number of FTR1 in *L. corymbifera* compared to *R. oryzae* (one copy) suggests more efficient employment of this pathway and, probably, optimisation of the different copies to different environmental conditions in *L. corymbifera*. To investigate the expression of iron-uptake genes under iron limited conditions we added the iron chelator bathophenanthrolinedisulfonic acid (BPS) to overnight cultures of *L. corymbifera* (200 µM final concentration) and analysed gene expression via RNA sequencing (see Material and Methods for details). Consistent with this hypothesis only one of the copies of FTR1 (LCor01036.1) was up-regulated under iron limitation in the gene expression experiments while another copy (LCor06326.1) was moderately down-regulated (Figure 5 A). In addition, all multicopper oxidases which are co-localized with FTR1 were regulated in the same manner as the corresponding FTR1 gene and expressed at comparable levels (Figure 5 A). The two remaining copies (LCor00518.1, LCor04103.1) were expressed constitutively at low levels and may be either specific for other conditions or generally silenced. In addition to FTR1 and multicopper oxidases, ferric reductases are key elements in the reductive pathway of iron acquisition. Two of the three ferric reductases were up-regulated under iron limitation (LCor07115.1, LCor11373.1), whereas the third one was constitutively expressed (Figure 5 B).

Besides the role of the reductive pathway little is known about the iron uptake systems in mucoralean pathogens. Our analysis uncovered the presence of additional genes involved in iron uptake including zinc/iron permeases, heme oxygenases and siderophore transporters (Table 2). Heme utilization may contribute to growth within the host, since mucoralean pathogens rapidly invade blood vessels [3] and may use hemoglobin as iron source [60]. Accordingly, one of the heme oxygenases (LCor09772.1) was strongly up-regulated under iron limitation (Figure 5 B). All available mucoralean genomes, including *L. corymbifera*, lack non-ribosomal peptide synthetases (NRPSs) and are therefore unable to produce hydroxamate siderophores. Instead they produce polycarboxylate siderophores (rhizoferrin), which have a much weaker binding activity compared to hydroxamate siderophores and are produced by direct fermentation [64], [65]. In addition, mucoralean fungi are also able to utilize deferoxamine, a bacterial siderophore which is used as an iron-chelator in human therapy [66], [67]. Interestingly, zygomycetous species have been shown to live in close relationship with bacteria, including cases containing bacterial endosymbionts indicating that xenosiderophores might play a role in the development of siderophore uptake systems [68], [69]. However, *L. corymbifera* has also been shown to be a potent producer of siderophores itself [70]. Under iron-limitation the putative siderophore transporter of *L. corymbifera* (LCor01340.1) was up-regulated (Figure 5 B) supporting the role of siderophores in the iron acquisition of mucoralean fungi. Interestingly, based on the expression data we found an additional gene (LCor00410.1) that may be involved in the siderophore metabolism of *L. corymbifera*, containing functional domains which are typical for genes involved in regulation and synthesis of siderophores in bacteria (lucA/lucC PF04183 and FhuF PF06276). Thus, the gene may encode a novel candidate for a regulator of siderophore biosynthesis in mucoralean fungi.

**Table 2:** Iron uptake genes in the *L. corymbifera* genome.

Pathway	Iron uptake gene	Number of genes
Reductive pathway	FTR1	4
	multicopper oxidase	8
	ferric reductase	3
Low affinity iron uptake	zinc/iron permease	6
Siderophore uptake	siderophore transporter	1
Heme utilization	heme oxygenase	2
Iron storage	ferritin	2

Although fungi generally lack ferritin as intracellular iron storage, ferritin has been found in several mucoralean species [62], [71]. Based on orthology searches, ferritin genes could be identified in all mucoralean genomes. In addition, domain search in *Spizellomyces punctatus* and *Allomyces macrogynus* (Origins of Multicellularity, BROAD) revealed also the presence of potential ferritin genes. Since these two groups represent the most basal fungal lineages, apart from the highly derived microsporidians [72], ferritin seems to have been lost in some microsporidians and the higher fungi. Interestingly, in higher fungi, the loss of ferritin coincides with the appearance of sidA, an important gene in hydroxamate siderophore production (Figure S6). Siderophores are known to serve as intracellular iron storages in several Asco- and Basdiomycota species [73], [74]. Thus, a plausible hypothesis is that maintenance of ferritin was not necessary in derived fungi due to the gain of importance of siderophores. The expression of the two ferritin genes (LCor08103.1, LCor11038.1) was slightly decreased under iron-limitation (∼1.6×, Fig. 5B). Nothing is known about the dynamics and functions of fungal ferritins. The slight decrease may be sufficient to stabilize free iron concentrations in the cytoplasm.

Only three transcription factors were up-regulated under iron depleted conditions (Figure S7). Interestingly, one of these genes (LCor08192.1) shows similarities to GATA type regulators which are known to be involved in the adaptation to iron limitation in higher fungal pathogens (e.g. *A. fumigatus and Histoplasma capsulatum*) [75], [76]. Thus, this transcription factor may represent a key regulator in iron acquisition, and therefore an important virulence factor of *L. corymbifera*. A second transcription factor (LCor01236.1) resembles CRZ1, which is a calcineurin regulated TF and indicates a possible involvement of the calcineurin pathway in the adaption to iron limitation.

#### b) Secreted proteases

Besides the iron uptake systems, hydrolytic enzymes like proteases are known as important virulence factors in fungal pathogens, e. g., *R. microsporus* and *C. albicans* [77]–[79]. In addition, gene expansion of secreted proteases was observed in the *R. oryzae* genome [28]. The genome of *L. corymbifera* contains a total of 413 predicted proteases representing 3.3% of all genes comparable to the situation in *R. oryzae* which contains 630 proteases (3.6% of all genes). However, the number of secreted proteases differs between the two species, moreover, in *L. corymbifera* the relative amount of secreted proteases is nearly twice as high as in *R. oryzae*: in *L. corymbifera* 13% (53) of the proteases are predicted to be secreted while in *R. oryzae* this number reaches only 7% (44). The most important classes of secreted proteases are serine and aspartate proteases representing 55% and 36% of total secreted proteases, respectively (Table 3). Comparison of secreted aspartic proteases (SAP) of *R. oryzae* revealed an enrichment of SAPs compared to other fungal genomes [28]. However, the number of SAPs is comparable in *L. corymbifera* (24) and *R. oryzae* (28) indicating that the presence of a higher number of these enzymes is a general feature of mucoralean pathogens. Several secreted proteases were activated under infection-related stress conditions (iron depletion, hypoxia). While iron depletion affected mainly the expression of aspartic and serine proteases, hypoxic conditions induced the expression of serine-, metallo- and some aspartic proteases.

**Table 3:** Protease families in the *L. corymbifera* genome.

	Proteases (% of total proteases)	Secreted (% of secreted proteases)
Aspartate	60 (14.5)	19 (35.8)
Cysteine	68 (16.5)	2 (3.8)
Metallo	146 (35.4)	3 (5.6)
Serine	124 (30)	29 (54.7)
Threonine	14 (3.4)	0
Unknown	1 (0.2)	0
Total	413	53

#### c) Transcription factors

The *Lichtheimia* genome encodes 768 putative transcription factors (TFs) representing 6.2% of total genes. This amount is comparable to the situation in *R. oryzae* (6.4% of total genes) but higher than the average content of TFs in other fungi (4.5%) [80]. Basic BLASTp analyses showed that 37 of the TFs (4.8% of total TFs) are specific for *L. corymbifera*.

The TFs were assigned to 53 families of DNA-binding domains (based on the InterProScan predictions). The great majority of these families have been described previously in fungal species [81]. However, 4 families have not been found in true fungal species before (putative representatives of 2 families, PF01167 (Tubby) and PF02319 (TDP), were predicted in microsporidia; the other 2 were described in plants and bacteria). Of these “new” TFs one (PF03106, DNA-binding WRKY domain), represented in 7 *Lichtheimia* proteins, has been previously described only in plants. Interestingly, in plants these TFs are numerous and have diverse functions, including pathogen defense [82]. In *L. corymbifera* we found three of the seven members differentially regulated under hypoxic conditions (down: LCor09690.1; up: LCor02851.1, LCor08197.1) indicating a function in the stress response of this species (Figure S7).

Of 6 fungal-specific TF families [81], 2 families are not predicted in *L. corymbifera* genome, namely PF04769 (mating-type protein MAT a1) and PF02292 (APSES domain). The lack of MAT gene is expected because mating in Mucorales is regulated via sex plus and sex minus HMG transcription factors [83]. The absence of APSES domain may be compensated by the APSES-type DNA binding domain PF04383.

It is also noteworthy that the traditional proportion of the Zn fingers Zn(2)Cys(6) (Zn cluster) and Cys(2)His(2) is inverted in *Lichtheimia*. In all fungi observed so far, the Zn clusters are more abundant than C2H2 TFs, in fact they are normally the most numerous in the fungal genomes. In *Lichtheimia*, on the contrary, the number of C2H2 Zn fingers is nearly 1.5 times larger than the number of Zn clusters.

Comparative phylome-based analysis reveals several expanded TF families in *L. corymbifera* including MADS box TFs with 11 representatives instead of the usual 1–4 members. MADS box genes are known to play a role in a variety of functions (e.g, cell cycle, stress response, development [84]). Presumably, the expansion of the MADS box genes in *Lichtheimia* was accompanied by the delegation of some functions from other TFs or even neofunctionalization. The functional basis for such expansion as well as the potential roles of these TFs cannot be elucidated from their primary structure, because MADS box genes are not conserved except for the MADS domains. But at the epxressional level, we could find significant up-regulation of two of the 11 MADS box TFs (LCor03918.1, LCor08105.1). Thus, the copies do not seem to have completely overlapping functions.

Analysis of the phylomes allowed us to detect another exciting expansion, which is evidently characteristic for all Mucorales: the duplication of TBP, TATA binding protein (PF00352). As it has been recently shown for higher eukaryotes, core promoter recognition factors can be involved in modulating gene- and cell-type-specific programs of transcription, such as tissue differentiation, development, etc. [85]. These new functions are associated with a gene duplication of the TBP, resulting in TRF2 (and other) factors, which are highly similar to TBP but do not bind the TATA box. In fungi, the event of TBP duplication is exceptionally rare. A survey of all so far sequenced genomes revealed only 4 examples of such duplication: 3 in Ascomycetes/Sordariomycetes (*Chaetomium globosum*, *Grosmannia clavigera* and *Podospora anserina*) and 1 in the basidiomycete *Laccaria bicolor*. In contrast, in Mucorales all 4 considered species possess 2 copies of the TBP gene. It can be supposed that the duplicated TBP-like factors may play an additional role in condition-specific responses and thus may be of interest as potential virulence factors.

Temperature tolerance is an essential prerequisite for the infection of warm-blooded animals and was shown to be connected to the virulence of *Lichtheimia* species [25], [86]. The genome was surveyed for the presence of heat shock transcription factors (HSF). The total number of these TFs in *L. corymbifera* genome is 24, which is the highest number among all so far investigated fungi. This is in accordance with the known tolerance of *L. corymbifera* to high temperatures [13], [14]. However, it seems that this family expansion is not a specific trait of *Lichtheimia* but is characteristic for all Mucorales. Interestingly, HSF genes were also up-regulated under hypoxic conditions indicating additional functions of the different members of the HSF family in the response to different stresses and growth conditions.

It is curious that additionally to the abundant heat shock factors also a cold shock TF (PF00313) was found, which was not previously described in fungi. This can explain why *Lichtheimia*, although it does not grow at low temperatures, can tolerate cold as it was shown to survive periods of more than 5,000 years in ice [87].

### The importance of the *L. corymbifera* genome for studying the infection biology of mucoralean pathogens: Concluding remarks

The genome of *L. corymbifera* represents the first insight into the genome structure of basal mucoralean pathogens. Despite the growing recognition of Mucorales as life-threatening clinically important human pathogens, little is known about the virulence traits of these fungi. The high dissimilarity between *L. corymbifera* and the other sequenced mucoralean pathogens *R. oryzae* and *M. circinelloides* in both evolutionary and functional sense underlines the importance of additional genome projects.

This study revealed a high proportion of duplicated and expanded genes in the *L. corymbifera* genome comparable to the situation in *R. oryzae*. However, clear evidence for a WGD can be detected only for *R. oryzae*, but not for *L. corymbifera* indicating that additional mechanisms contribute to the higher incidence of duplicated genes in mucoralean fungi. Tandem repeats seem to be an important source for gene duplication in *L. corymbifera* and may explain the rapid development of lineage-specific gene duplication and expansion in mucoralean fungi. Several species-specific gene duplications point at potential virulence traits including iron uptake genes, hydrolytic enzymes and genes which may contribute to resistance against antifungal agents like azoles (cytochrome P450 gene expansion). In contrast, alternative splicing does not seem to play an important role in the generation of orthologs and the adaption to stress conditions.

Based on these results, we postulate a relationship between genome fluidity by the generation and retention of additional gene copies and dynamics of adaptation to new environments. Higher genome flexibility results in a higher likelihood for a saprobic zygomycete to become a pathogen.

In addition we were able to shed light on the genes involved in iron uptake, which is a crucial step for virulence and thus for the development of an infection. We could identify additional genes which might be involved in iron-uptake besides the known virulence factor FTR1 *L. corymbifera* including transcription factors, siderophore transporters and a potential regulator involved in siderophore biosynthesis that has not been described in mucoralean fungi.

Our data represent a valuable resource for future research and the understanding of infection-associated mechanisms of mucoralean pathogens.

## Materials and Methods

### Genome sequencing and assembly

A combination of Illumina and 454 sequencing was used for the *L. corymbifera* genome. A shotgun library and an 8 kb paired-end library were created and sequenced on a half plate on a Roche GS FLX Titanium each resulting in 1,168,226 shotgun reads (505,023,982 nt) and 519,989 paired-end reads (76,603,029 nt). In addition, a standard paired-end read library was prepared and sequenced in one channel Illumina HiSeq2000 (100 bp paired-end reads) resulting in 264,907,616 raw reads (26,490,761,600 nt) and 12,614,650 filtered and downsampled reads (1,261,465,000 nt). The 454 reads were separately assembled using Newbler (454 Life Sciences) and Mira [88] and both assemblies were unified using minimus2 [89]. The Illumina reads were used to solve homopolymeric regions using Nesoni (http://bioinformatics.net.au/software). This approach resulted in a total of 1,214 contigs (≥500 nt) with a total of 41,405,106 nt and a N50 of 66.718 nt. Finally, the contigs were mapped on Newbler predicted scaffolds using MUMmer [90] resulting in 209 scaffolds with a total length of 33.6 Mb (for statistics refer to Table 1). The raw DNA-seq reads and the resulting genome assembly is available at EMBL under the study accession number PRJEB3978 (http://www.ebi.ac.uk/ena/data/view/PRJEB3978).

### Detection of transposable elements

Scaffolds of *L. corymbifera* were searched for repeats by Repbase and the server version of Censor [91], [92] (http://www.girinst.org/censor/index.php) using the eukaryotic repeat database.

### K-mer analysis

The analysis was performed on the Ilumina reads with an algorithm described in the potato genome paper [31]. The algorithm was used to write a custom perl program. Based on the fastq data of the Illumina reads k-mers of 41, 59, 69, and 79 nt were detected and analyzed. Component estimation was done manually in R.

### Non-coding RNA prediction, synteny and phylogeny

A local version of tRNAscan-SE v.1.23 [93] with parameters –omlfrF was used for the detection of tRNAs. Ouput files are supported in the supplemental material (http://www.rna.uni-jena.de/supplements/lichtheimia/index.html). With RNammer -S euk -m lsu,ssu,tsu -gff (v.2.1) [94] rRNAs were detected. The 1973 ncRNA classes currently available at RFAM (v.10.1) [95] were downloaded for homologous search. These classes were predicted with (I) BLAST (v.2.2.25) [96] with an E-value<10^−4^ (II) with infernal [97] using covariance models from RFAM and (III) by hand as indicated in main text. Genes discovered in the reads only were found with rnabob [98] in combination with various programs of the RNAViennaPackage v.2.0.2 (http://www.tbi.univie.ac.at/~ivo/RNA/). All ncRNA genes are available at the supplemental material in gff and fasta format (http://www.rna.uni-jena.de/supplements/lichtheimia/index.html). Additionally, sequence-structure-alignments for each RFAM-ncRNA class in stockholm format are provided. Motif search in promoter regions of polymerase III transcripts was performed with MEME (v.4.8.1) [99], rnabob and by hand. Synteny analysis: for all of our identified ncRNA positions in *L. corymbifera* and *R. oryzae*, five direct upstream and downstream located genes and their function were extracted, according to protein-annotation files. Pairwise alignments of syntenic proteins with a) -p blastn and b) -p tblastn and a minimum E-value of E<10^−4^ were performed. For ncRNA-phylogeny reconstruction the best scored ncRNA per ncRNA family was joined, which was identified in all species, except 18S and 28S rRNA, and *S. pombe* used as outgroup. A multiple alignment was created by Mafft with the L-INS-i method, 1000 iterations as module in the EPoS framework for phylogenetic analysis [100]. Out of this alignment we constructed a Neighbour Joining Tree (Kimura correction model, 1000 bootstrap replicates) and Mr. Bayes (v.3.1.2; two runs with each four chains and 5,000,000 generations).

### Prediction of protein-coding genes and functional annotation

Evidence-driven gene prediction was performed using AUGUSTUS v2.7 [37] using the gene models from *Rhizopus oryzae* prediction was supported by the incorporate pooled Illumina RNA-seq data from three biological replicates of three different physiological conditions (control, hypoxia, iron depletion) sequenced on Illumina HiSeq 2000. After the raw RNA-Seq data were quality trimmed- using btrim [101], the data were pooled and mapped using the splice-junction mapper tophat2 [102]. From this mapping data the AUGUSTUS protocol (http://bioinf.uni-greifswald.de/bioinf/wiki/pmwiki.php?n=IncorporatingRNAseq.Tophat) was followed to create hints for gene structures in an iterative manner. Finally, the hints were incorporated during the AUGUSTUS predcition based on the *Lichtheimia* genome using the metaparameters of *R. oryzae*. For functional annotation predicted protein-coding genes were analyzed by BLASTp in BLAST2GO [38] with a minimum E-value of E≤10^−25^ and a HSP length cut-off of 33 amino acids. Conserved domains were identified using the InterProScan function of BLAST2GO and GO mapping was performed based on the BLAST and InterProScan results. Genome annotations are available at the ENA under the study accession number PRJEB3978 (http://www.ebi.ac.uk/ena/data/view/PRJEB3978).

### Detection of differentially expressed genes under infection-related conditions

Paired-end RNA-seq data for three biological replicates of two infection-associated conditions (i.e., iron-depletion and hypoxia) and a control treatment was obtained.

*L. corymbifera* was grown on SUP agar [103] plates for 7 days at 37°C. Spores were washed off with sterile PBS, washed with PBS and counted using a Thoma chamber. Erlenmeyer flasks (500 ml) containing 100 ml of chemical defined medium (1.7 g/l YNB w/o amino acids and ammonium sulphate, 20 g/l Glucose, 5 g/l ammonium sulphate, 50 mg/l arginine, 80 mg/l aspartic acid, 20 mg/l histidine, 50 mg/l isoleucine, 100 mg/l leucine, 50 mg/l lysine, 20 mg/l methionine, 50 mg/l phenylalanine, 100 mg/l threonine, 50 mg/l tryptophane, 50 mg/l tyrosine, 20 mg/l valine) [60] were inoculated with 10^7^ spores and grown for 16 h at 37°C under shaking. Afterwards (i) cultures were grown for additional 2 h under these conditions, (ii) the iron chelator bathophenanthrolinedisulfonic acid (BPS, Sigma) was added to a final concentration of 200 µm and cultures were incubated for additional 2 h under previous conditions or (iii) cultures were subjected to hypoxic conditions (1% oxygen, 5% CO_2_) and incubated for 2 h at 37°C under shaking. The mycelium was separated from the medium using a miracloth filter (Millipore) and immediately frozen in liquid nitrogen. For RNA isolation the mycelium was grounded using mortar and pestle under liquid nitrogen and total RNA was isolated using the RNAeasy Plant kit (Quiagen) according to the manufacturer's instructions.

Sequencing was performed using Illumina HiSeq 2000. Raw reads were quality-filtered using btrim [101] and mapped to the genome using tophat2 [102] (parameters: –no-discordant –no-mixed –b2-very-sensitive –max-intron-length 5000). Differentially expressed genes were identified with EdgeR [104] which also adjusted obtained p-Values for multiple testing. Transcripts with an absolute fold-change≥2 and an adjusted p-Value≤0.01 were considered differentially expressed. Results are available in Table S7.

### Phylome reconstruction

The phylome, meaning the complete collection of phylogenetic trees for each gene in a genome, was reconstructed for the genome of *L. corymbifera*. 24 other fungal species were included in the reconstruction. A rough draft of the proteome of *Mortierella alpina* ([26]; PUBMED ID:22174787) was predicted using AUGUSTUS [32] due to the lack of a publicly available proteome. The phylome was reconstructed using an automated pipeline previously described in [39]. Briefly, for each protein in the *L. corymbifera* genome a Smith-Waterman search was performed against the fungal proteome database. Results were filtered using an e-value cut-off E<1e^−5^ and a continuous overlapping region of 0.5. At most 150 homologous sequences for each protein were accepted. Homologous sequences were then aligned using three different programs: MUSCLE v3.8 [105], MAFFT v6.712b [106], and kalign (http://www.biomedcentral.com/1471-2105/6/298/)]. Alignments were performed in forward and reverse direction (i.e. using the Head or Tail approach [107]), and the 6 resulting alignments were combined with M-COFFEE [108]. This combined alignment was trimmed with trimAl v1.3 [109] (consistency-score cut-off 0.1667, gap-score cut-off 0.9). Trees were reconstructed using the best-fitting evolutionary model. The selection of the model best fitting each alignment was performed as follows: a Neighbour Joining (NJ) tree was reconstructed as implemented in BioNJ [110]; the likelihood of this topology was computed, allowing branch-length optimization, using 7 different models (JTT, LG, WAG, Blosum62, MtREV, VT and Dayhoff), as implemented in PhyML v3.0 [111]; the model best fitting the data, as determined by the AIC criterion [112], was used to derive ML trees. Four rate categories were used and invariant positions were inferred from the data. Branch support was computed using an aLRT (approximate likelihood ratio test) based on a chi-square distribution. Resulting trees and alignments are stored in phylomeDB [39] (http://phylomedb.org), with the phylomeID 245. Trees were scanned using ETE v2 [46].

### Orthology prediction

Orthologs between *L. corymbifera* and the other species included in the phylome were based on phylogenies obtained during phylome reconstruction. A species-overlap algorithm, as implemented in ETE v2 [46], was used to infer orthology and paralogy relationships. Briefly the algorithm decides whether a node in a tree is a speciation of a duplication node depending on the overlap of the species branching from the node. Overlap between those species will indicate a duplication node. Otherwise a speciation node will be considered.

### Species tree reconstruction

The species tree was build using a concatenation method. 58 single-copy proteins that appeared in at least 21 of the 25 genomes were selected. After concatenation, the alignment was trimmed using trimAl [109]. Columns with more than 50% of gaps were removed. A conservation score of 50% of the alignment was used. The final alignment contained 46,793 positions. The tree was reconstructed using phyML [111]. LG model [113] was selected and a 4-categories GAMMA distribution was used. Bootstrap was obtained by creating 100 random sequences using SeqBoot from the phylip package. A tree was then reconstructed for each sequence and the consensus tree was inferred using phylip. All the nodes in the species tree had a bootstrap of 100. Additionally a species tree based on the super-tree reconstruction program DupTree [42] was reconstructed. The input contained the 9,478 trees obtained during phylome reconstruction. Both species trees showed a similar topology. The only difference pertained to the position of *S. pombe*. In the concatenated tree it appeared grouped with *S. cerevisiae* while in the super-tree it appeared in its correct position at the base of Ascomycota. This difference was collapsed into a multifurcation for the tree in figure 1.

### Detection of conserved regions

For the detection of conserved regions, all genomes were modeled as strings of integers. BLAST analyses [96] were performed for all proteins in the four mucoralean genomes all-against-all, with an E-value threshold of 0.1. Homology families IDs were assigned to the protein-coding genes using GhostFam [114] with default parameters. Genomes were transformed into strings of gene IDs, which were then used as input for the reference gene cluster implementation in Gecko2 [43], [44]. The two parameters for the algorithm were the minimum size of the reference cluster/hypothetical conserved region “s” and the maximal distance “δ” (insertion or deletion of a gene). For every hypothetical gene cluster larger than s on the reference genome, all other genomes were tested for approximate occurrences of this reference gene cluster. The *L. corymbifera* genome was used as a reference genome and searched for gene clusters with parameters s = 3 (minimum size of the reference gene cluster) and δ = 0 (number of insertions and deletions), s = 4/δ = 1, s = 5/δ = 2, s = 6/δ = 3 and s = 7/δ = 4. Results of the different filter settings were combined and overlapping clusters were eliminated. Local rearrangements and duplications within the cluster occurrences were not punished. All regions that had approximate occurrences in at least one other genome were reported. If multiple occurrences did intersect, only the best scoring one was reported.

### Detection of duplicated regions (segmental duplications)

To detect duplicated regions in the mucoralean species, each genome was analysed individually by using the single contigs as reference. As for the detection of conserved regions, the same homology assignment and parameters of s = 5 and δ = 2 were used. All regions with approximate occurrences in at least one other contig or the reference contig were reported, unless they intersected.

### Detection of tandem duplications

Tandem duplications were defined by at least two genes assigned to the same GhostFam gene family and a maximum of three genes between the copies.

### Prediction of alternative splicing

Predicted transcripts of the genomes were separated in alternative splicing events by Astalavista [24]. Events of predicted transcripts that contain splice-junctions have been confirmed by the number of split-mappings that confirm each of the exon-exon junctions (Table S8). For a read to support a splice-junction, the left part of the read was required to be included in one exon, and the right part had to be included in the other exon of a splice junction, with the first/last position before/after the split matching exactly the position of the predicted intron.

### Genome resources

Genome data of *Aspergillus fumigatus* [115], *Aspergillus nidulans* [116], *Batrachochydrium dendrobatidis*, *Cryptococcus neoformans*, *Encephalitozoon cuniculi* [117], *Rhizopus oryzae* [28], *Paracoccidioides brasiliensis*, *Schizosaccharomyces pombe* [118], *Nosema ceranae* [119], *Nematocida parisii* [120], *Puccinia graminis* [121], *Ustilago maydis* and *Coprinus cinerea* [122] are genome sequencing projects of the Broad Institute of Harvard and MIT (http://www.broadinstitute.org/) (see Table S9 for detailed citations). *Phycomyces blakesleeanus*, *Phanerochaete chrysosporium* [123], *Laccaria bicolor* [124], *Mucor circinelloides*, *Nematostella vectensis* [125], *Monosiga brevicollis* [126] and *Serpula lacrymans* [127] genomic data were obtained from Joint Genome Institute (JGI). These sequence data were produced by the US Department of Energy Joint Genome Institute http://www.jgi.doe.gov/ in collaboration with the user community. The genomes of *Homoloaphlyctis polyrhiza* [128] and *Mortierella alpina* [26] were obtained from Genbank (*Hp*: PRJNA68115; *Ma*: PRJNA41211). The *Neurospora crassa* genome [129] was obtained from UniProt reference genomes. The *Saccharomyces cerevisiae* genome was obtained from *Saccharomyces* Genome database (SGD) (see Table S9) [53].

## Supporting Information

Figure S1 K-mer frequency distribution for *Lichtheimia corymbifera*. The k-mer frequency distribution (black line) was calculated for all k-mers of length of 59, i.e. for all possible 59-mers derived from the original Illumina/Solexa reads. The number of k-mers (y-axis) is plotted against the frequency at which they occur (x-axis). The distribution shows a main peak (shaded in light gray) and a steep rise to the left (shaded in dark gray). This left-most rise of k-mers at lower frequencies represents mostly k-mers with randomly occuring sequencing errors. The main peak represents k-mers derived from (putatively) correct sequencing reads. This main peak can be dissected into three normal distributions (red, blue and orange lines) the sum of which (green line) matches the observed distribution (black line). The three component distributions represent the ‘homozygous’ part of the genome (blue line, major component), the ‘heterozygous’ part of the genome (red line), and most likely some repeat regions that make up a minor proportion of the observed k-mers (orange line). Component estimation was done manually in R. The component distributions have the same variance (21), but different means (blue 116, red 65, orange 165) and proportions (blue 94%, red 4%, orange 2%).
(TIFF)

Figure S2 Structure of spliceosomal RNAs and ncRNA based phylogeny. (A) The last stem (IV) of *Lichtheimia* U11 snRNA is extended in comparison to other U11 snRNAs and to U1 snRNA. U2 snRNA folds into an expected secondary structure. In contrast, U12 snRNA shows an extended stem II, and misses the third stem (III). Stem IV/V is much shorter as in other known U12 snRNAs. U5 snRNA is used by both spliceosomes, with the general eukaryotic secondary structure. 2D structures were computed using RNAfold (RNA Vienna Package). Boxes indicate sm binding sites. Phylogeny of *L. corymbifera*, *M. circinelloides*, *P. blakesleeanus*, *R. oryzae* and *S. pombe* (outgroup) based on ncRNAs (except 18S and 28S rRNA). Alignment computed via Mafft L-INS-i with 1000 generations; Tree construction via (B) Neighbour Joining: Kimura: 1000 bootstrap replicates and (C) Mr. Bayes: two runs with each four chains and 5,000,000 generations.
(TIFF)

Figure S3 Tandem duplications in *L. corymbifera*. (A) Number and size of tandem duplication in the *L. corymbifera* genome. (B) Functional classes of genes in tandem duplications based on PFAM annotation. Asterisk indicates classes which are enriched in tandem duplications (Fisher test, P<0.05). (C) Gene family size comparison of *L. corymbifera* and *R. oryzae*. Gene families are indicated as larger in *Lichtheimia* (L>R), smaller in *Lichtheimia* (L<R) or as large as in *Rhizopus* (L = R).
(TIFF)

Figure S4 Expression of tandem duplicated genes. Tandem duplicated genes were analysed based on the RNA-seq data. Tandems were regarded as (i) not regulated if no copy in the cluster was up/down-regulated under the tested conditions, (ii) co-regulated if all copies in the clusters were up/down-regulated under at least one of the conditions, (iii) not co-regulated if one of the copies was differently regulated than the other(s), (iv) antithetically regulated if one copy was up- and the other down-regulated. Genes were regarded as differentially regulated if there was a two-fold change of expression and P<0.01 (edgeR).
(TIFF)

Figure S5 Alternative splicing in *L. corymbifera*. (A) Number and proportion of different classes of alternative splicing events based in evidence driven gene prediction (outer ring) and confirmed events (inner ring). (B) Proportion of AS genes where AS patterns were changed under stress conditions compared to control.
(TIFF)

Figure S6 Distribution of genes involved in iron uptake within the fungal kingdom. Orthologs of iron uptake genes were identified using the phylome of *L. corymbifera* (indicated in blue). If no ortholog was found BLASTp analysis was performed using the *L. corymbifera* protein sequence and an E-value E≤10^−10^ (indicated in pink). Intracellular iron storages besides ferritin are indicated as ‘s’ (siderophores) or ‘v’ (vacuolar) according to previous results (1 Silva et al., 2 Haas et al.). The presence of a sidA ortholog is indicated as “+”, the absence as “−” according to previous results (1 Silva et al., 2 Haas et al.).
(TIFF)

Figure S7 Differential expression of transcription factors under iron depletion and hypoxia. Bar charts on top represent TFs grouped according to their functional domains (domain combinations). Up- and down-regulated genes are indicated in red and green respectively. The bar chart on the bottom shows the total amount of TFs regulated under the conditions.
(TIFF)

Table S1 Sequencing statistic of the *L. corymbifera* genome and transcriptome.
(PDF)

Table S2 Transposable and repetitive elements in the *L. corymbifera* genome.
(PDF)

Table S3 Overview of ncRNAs found in basal fungi. op – Close/Part to Operon; pg – Pseudogene; Ror – *R. oryzae*; Lco – *L. corymbifera*; ? – candidate.
(PDF)

Table S4 Sequencing and mapping statistics of RNA sequencing.
(XLS)

Table S5 Signalling pathway components included in the study and orthologues identified in *L. corymbifera*.
(PDF)

Table S6 Classification and gene IDs of putative protein phosphatases in the *L. corymbifera* genome.
(PDF)

Table S7 RNA-Seq mapping and differentially expressed genes.
(XLS)

Table S8 Potential alternatively spliced genes in *L. corymbifera* and confirmation of alternative transcripts by RNA-Seq data.
(XLSX)

Table S9 Genomes used in this study.
(PDF)
