# Correction: Gene Expansion Shapes Genome Architecture in the Human Pathogen *Lichtheimia corymbifera*: An Evolutionary Genomics Analysis in the Ancient Terrestrial Mucorales (Mucoromycotina)

**DOI:** 10.1371/journal.pgen.1006491

**Published:** 2016-12-05

**Authors:** Volker U. Schwartze, Sascha Winter, Ekaterina Shelest, Marina Marcet-Houben, Fabian Horn, Stefanie Wehner, Jörg Linde, Vito Valiante, Michael Sammeth, Konstantin Riege, Minou Nowrousian, Kerstin Kaerger, Ilse D. Jacobsen, Manja Marz, Axel A. Brakhage, Toni Gabaldón, Sebastian Böcker, Kerstin Voigt

The following information is missing from the Funding section: This study was also funded by a grant from the Collaborative Research Center / Transregio 124 consortium “Pathogenic fungi and their human host: Networks of Interaction” (subprojects A1; B3; C5; Z1) to AAB, ES, KR, MM, IDJ and KV.
