# Supplementary figures and images for: Gene Expansion Shapes Genome Architecture in the Human Pathogen Lichtheimia corymbifera: An Evolutionary Genomics Analysis in the Ancient Terrestrial Mucorales (Mucoromycotina)
Source: PLoS Genet. 2014 Aug 14;10(8):e1004496. doi: 10.1371/journal.pgen.1004496 (PMC4133162; doi:10.1371/journal.pgen.1004496)

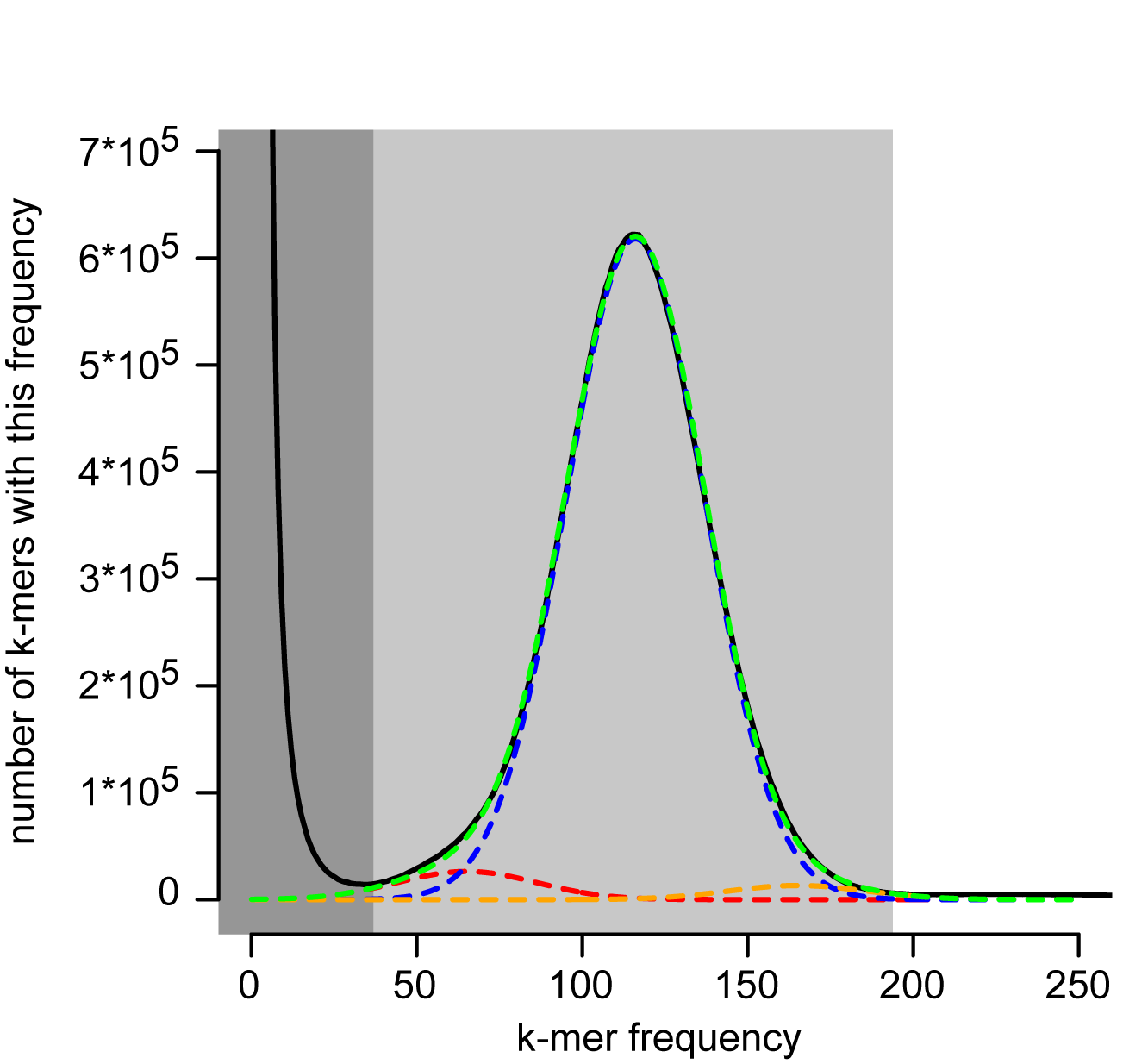

Supplement: Figure S1 — K-mer frequency distribution for Lichtheimia corymbifera. The k-mer frequency distribution (black line) was calculated for all k-mers of length of 59, i.e. for all possible 59-mers derived from the original Illumina/Solexa reads. The number of k-mers (y-axis) is plotted against the frequency at which they occur (x-axis). The distribution shows a main peak (shaded in light gray) and a steep rise to the left (shaded in dark gray). This left-most rise of k-mers at lower frequencies represents mostly k-mers with randomly occuring sequencing errors. The main peak represents k-mers derived from (putatively) correct sequencing reads. This main peak can be dissected into three normal distributions (red, blue and orange lines) the sum of which (green line) matches the observed distribution (black line). The three component distributions represent the ‘homozygous’ part of the genome (blue line, major component), the ‘heterozygous’ part of the genome (red line), and most likely some repeat regions that make up a minor proportion of the observed k-mers (orange line). Component estimation was done manually in R. The component distributions have the same variance (21), but different means (blue 116, red 65, orange 165) and proportions (blue 94%, red 4%, orange 2%). (TIFF) [file pgen.1004496.s001.tiff]

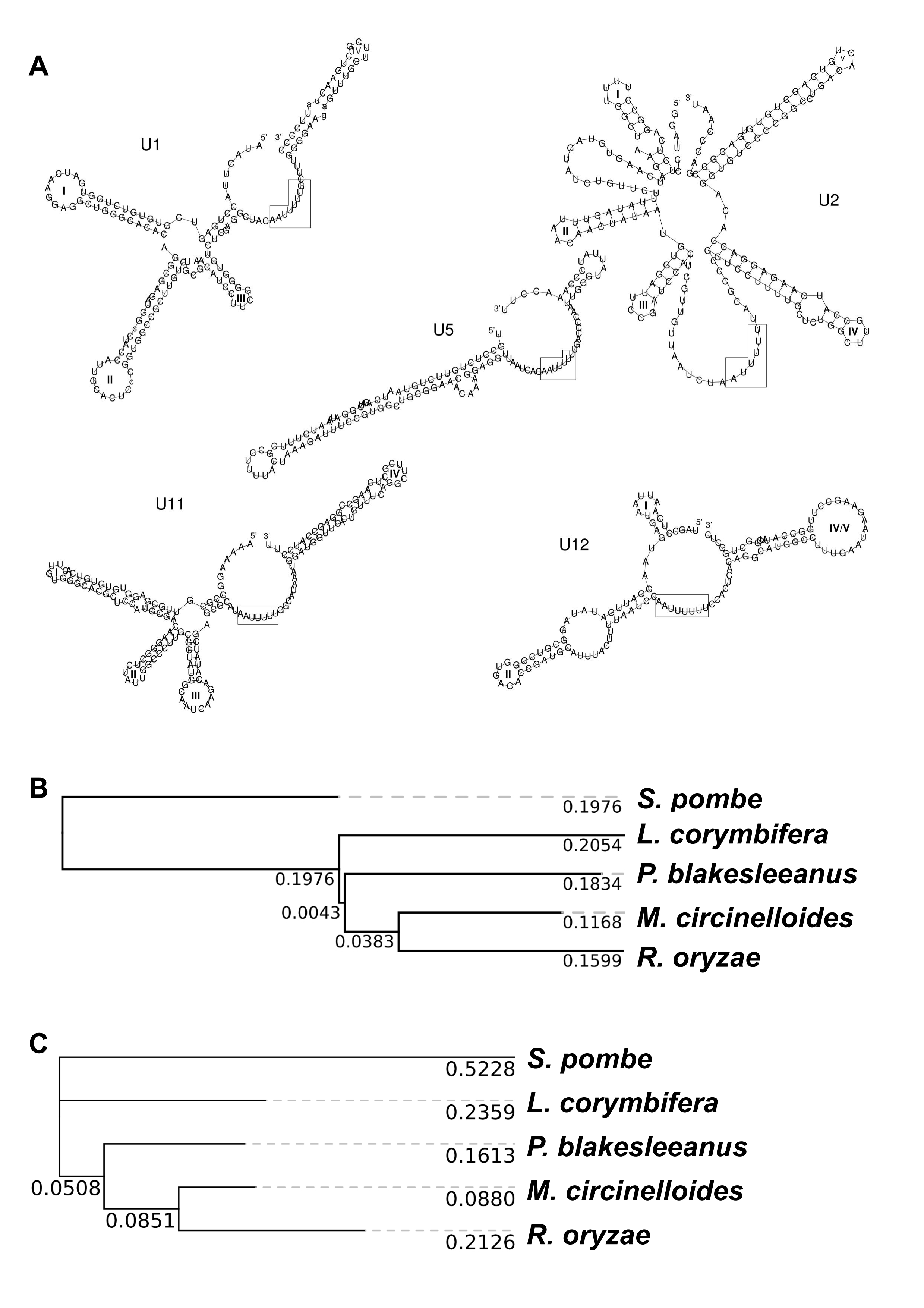

Supplement: Figure S2 — Structure of spliceosomal RNAs and ncRNA based phylogeny. (A) The last stem (IV) of Lichtheimia U11 snRNA is extended in comparison to other U11 snRNAs and to U1 snRNA. U2 snRNA folds into an expected secondary structure. In contrast, U12 snRNA shows an extended stem II, and misses the third stem (III). Stem IV/V is much shorter as in other known U12 snRNAs. U5 snRNA is used by both spliceosomes, with the general eukaryotic secondary structure. 2D structures were computed using RNAfold (RNA Vienna Package). Boxes indicate sm binding sites. Phylogeny of L. corymbifera, M. circinelloides, P. blakesleeanus, R. oryzae and S. pombe (outgroup) based on ncRNAs (except 18S and 28S rRNA). Alignment computed via Mafft L-INS-i with 1000 generations; Tree construction via (B) Neighbour Joining: Kimura: 1000 bootstrap replicates and (C) Mr. Bayes: two runs with each four chains and 5,000,000 generations. (TIFF) [file pgen.1004496.s002.tiff]

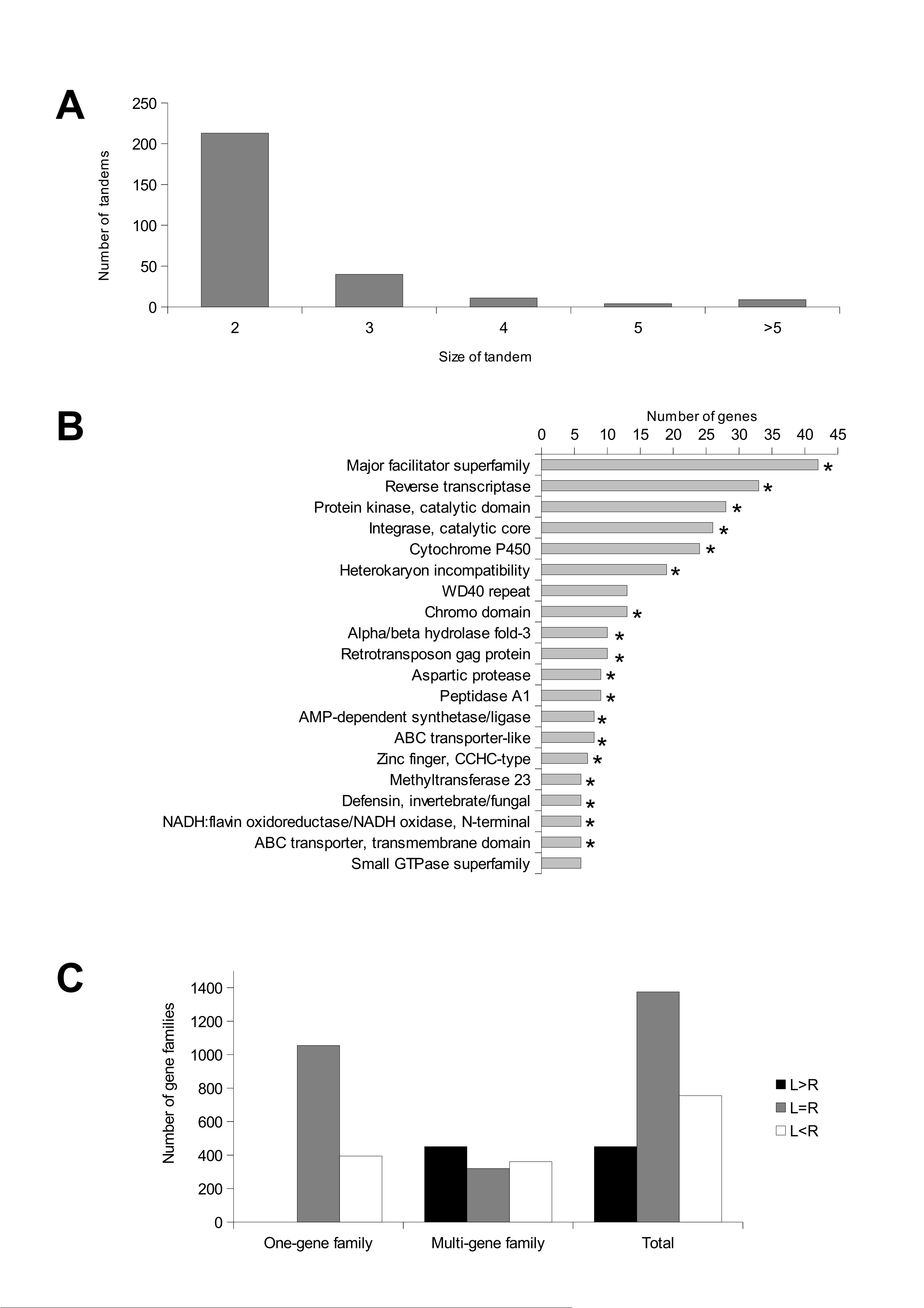

Supplement: Figure S3 — Tandem duplications in L. corymbifera. (A) Number and size of tandem duplication in the L. corymbifera genome. (B) Functional classes of genes in tandem duplications based on PFAM annotation. Asterisk indicates classes which are enriched in tandem duplications (Fisher test, P<0.05). (C) Gene family size comparison of L. corymbifera and R. oryzae. Gene families are indicated as larger in Lichtheimia (L>R), smaller in Lichtheimia (L<R) or as large as in Rhizopus (L = R). (TIFF) [file pgen.1004496.s003.tiff]

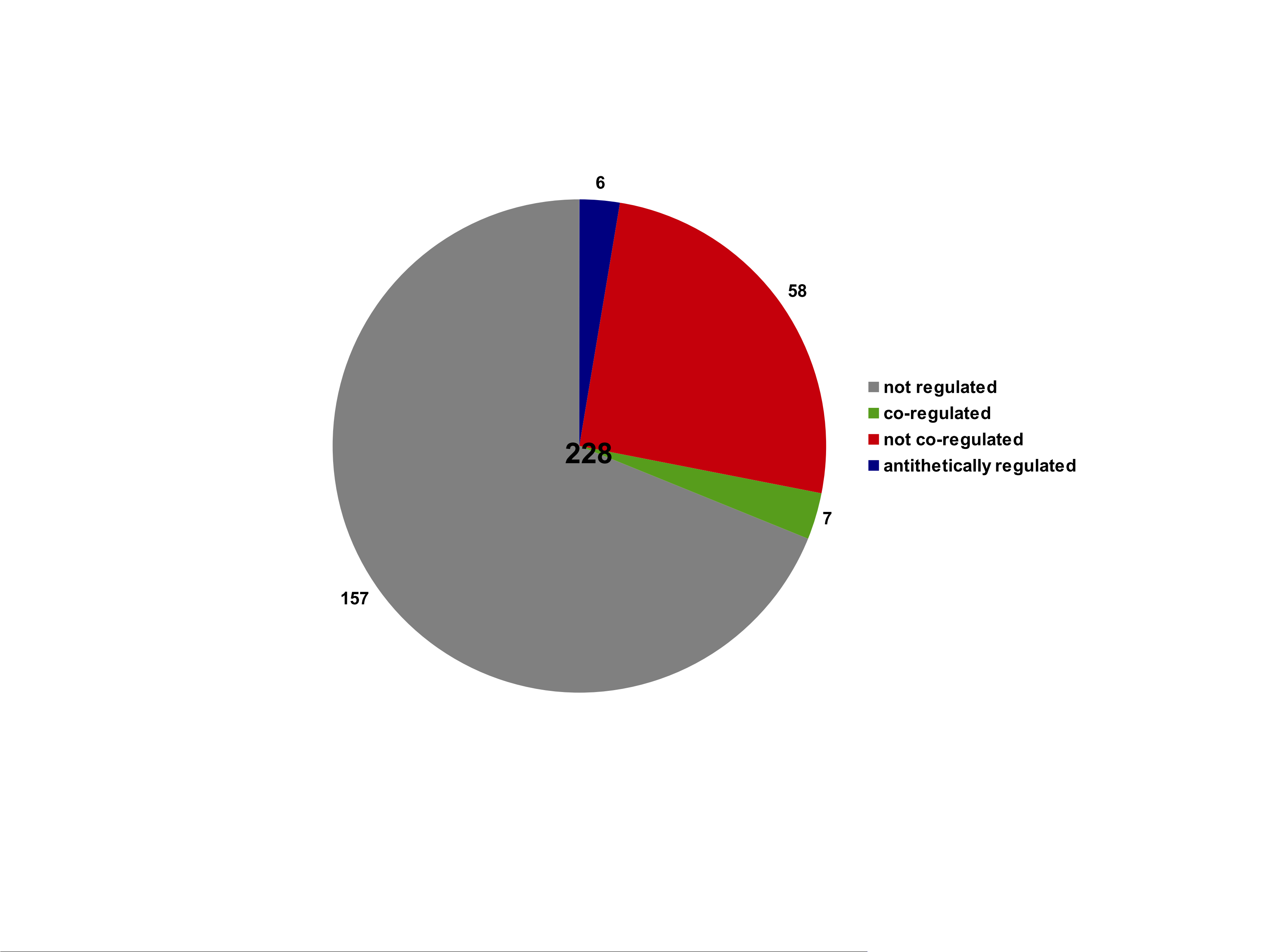

Supplement: Figure S4 — Expression of tandem duplicated genes. Tandem duplicated genes were analysed based on the RNA-seq data. Tandems were regarded as (i) not regulated if no copy in the cluster was up/down-regulated under the tested conditions, (ii) co-regulated if all copies in the clusters were up/down-regulated under at least one of the conditions, (iii) not co-regulated if one of the copies was differently regulated than the other(s), (iv) antithetically regulated if one copy was up- and the other down-regulated. Genes were regarded as differentially regulated if there was a two-fold change of expression and P<0.01 (edgeR). (TIFF) [file pgen.1004496.s004.tiff]

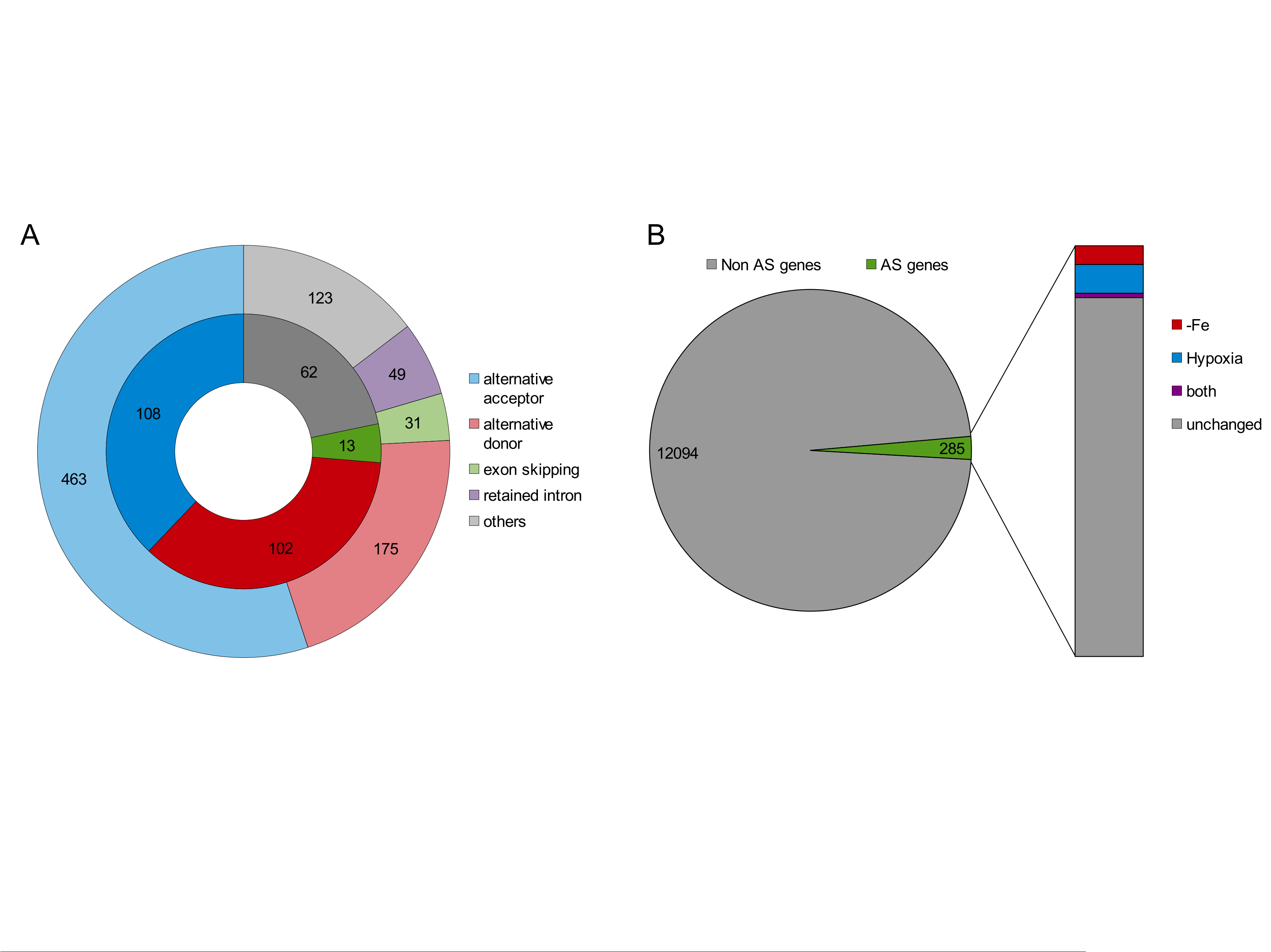

Supplement: Figure S5 — Alternative splicing in L. corymbifera. (A) Number and proportion of different classes of alternative splicing events based in evidence driven gene prediction (outer ring) and confirmed events (inner ring). (B) Proportion of AS genes where AS patterns were changed under stress conditions compared to control. (TIFF) [file pgen.1004496.s005.tiff]

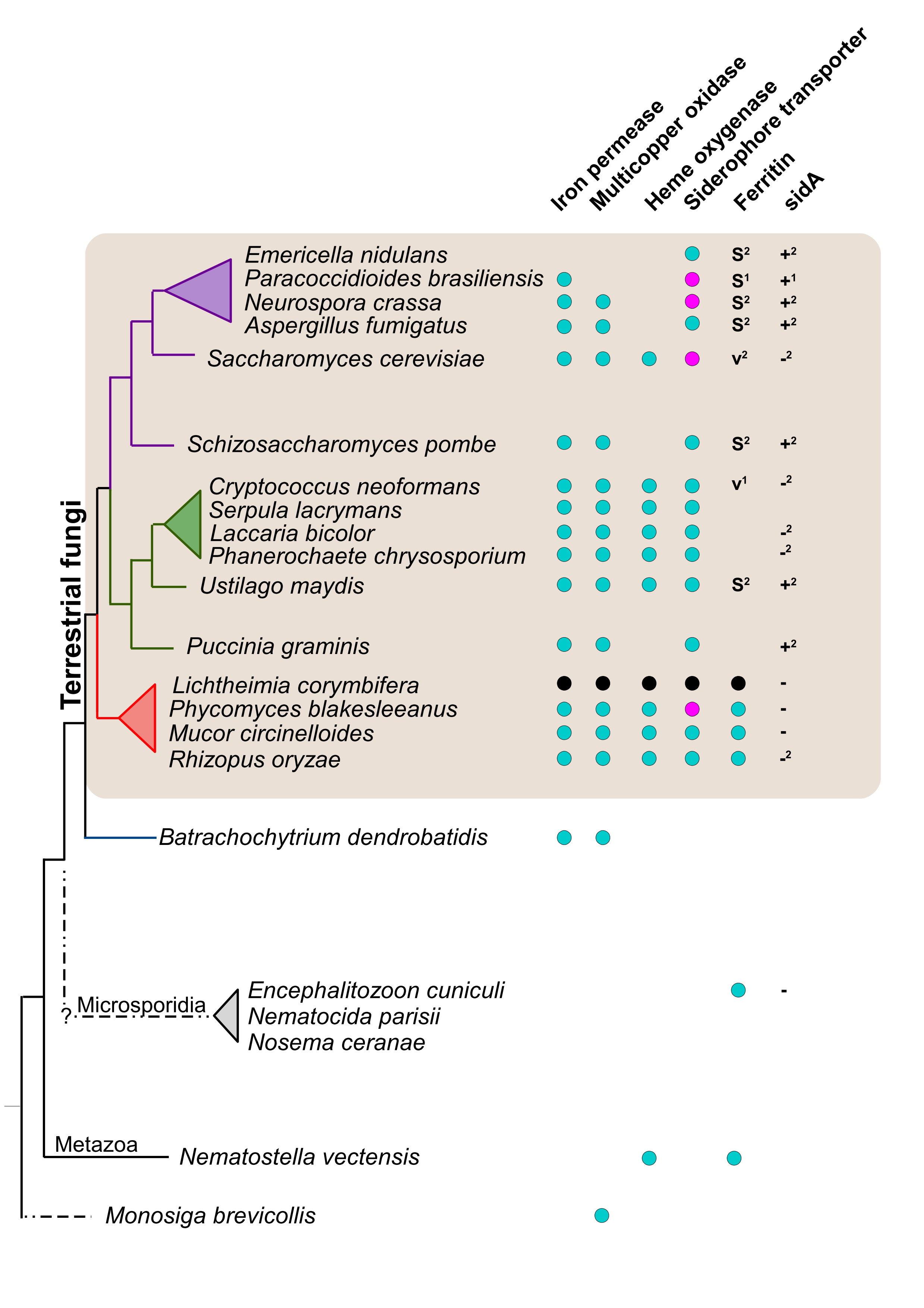

Supplement: Figure S6 — Distribution of genes involved in iron uptake within the fungal kingdom. Orthologs of iron uptake genes were identified using the phylome of L. corymbifera (indicated in blue). If no ortholog was found BLASTp analysis was performed using the L. corymbifera protein sequence and an E-value E≤10−10 (indicated in pink). Intracellular iron storages besides ferritin are indicated as ‘s’ (siderophores) or ‘v’ (vacuolar) according to previous results (1 Silva et al., 2 Haas et al.). The presence of a sidA ortholog is indicated as “+”, the absence as “−” according to previous results (1 Silva et al., 2 Haas et al.). (TIFF) [file pgen.1004496.s006.tiff]

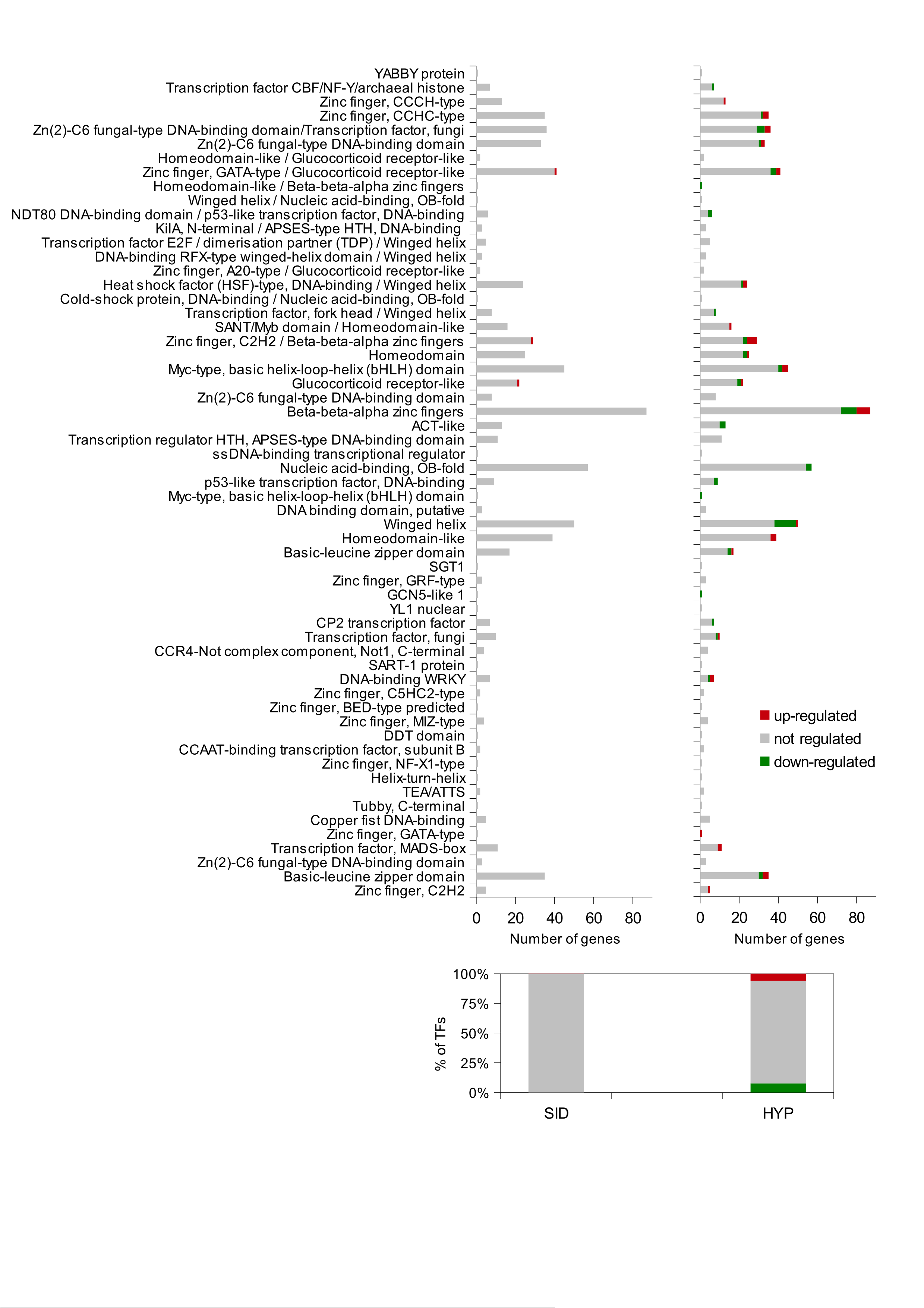

Supplement: Figure S7 — Differential expression of transcription factors under iron depletion and hypoxia. Bar charts on top represent TFs grouped according to their functional domains (domain combinations). Up- and down-regulated genes are indicated in red and green respectively. The bar chart on the bottom shows the total amount of TFs regulated under the conditions. (TIFF) [file pgen.1004496.s007.tiff]
