# Supplementary material for: Gene Expansion Shapes Genome Architecture in the Human Pathogen Lichtheimia corymbifera: An Evolutionary Genomics Analysis in the Ancient Terrestrial Mucorales (Mucoromycotina)
Source: PLoS Genet. 2014 Aug 14;10(8):e1004496. doi: 10.1371/journal.pgen.1004496 (PMC4133162; doi:10.1371/journal.pgen.1004496)
Supplement: Table S1 — Sequencing statistic of the L. corymbifera genome and transcriptome. (PDF) [file pgen.1004496.s008.pdf]

**Roche 454**	
Number of Shotgun Reads	1,168,226
Number of Bases in Shotgun Reads	505,023,982
Number of Paired-End Reads	519,989
Number of Bases in Paired-End Reads	76,603,029
**Illumina**	
Number of Raw Paired-End Reads	264,907,616
Number of Bases in Raw Paired-End Reads	26,490,761,600
Number of Filtered Paired-End Reads	240,264,256
Number of Bases in Filtered Paired-End Reads	24,026,425,600
Number of downsampled Paired-End Reads	12,614,650
Number of Bases in downsampled Paired-End Reads	1,261,465,000
**Assembly**	
**Newbler**	
Number of large Contigs (≥500 bp)	1,936
Number of Bases in Large Contigs	35,683,536
N50 Size of Large Contigs	57,255
Largest Contig Size	226,585
**Mira**	
Number of large Contigs (≥500 bp)	2,117
Number of Bases in Large Contigs	37,540,862
N50 Size of Large Contigs	46,267
Largest Contig Size	158,689
**Minimus2**	
Number of large Contigs (≥500 bp)	1,214
Number of Bases in Large Contigs	41,405,106
N50 Size of Large Contigs	66,718
Largest Contig Size	308,811
