# Supplementary material for: Gene Expansion Shapes Genome Architecture in the Human Pathogen Lichtheimia corymbifera: An Evolutionary Genomics Analysis in the Ancient Terrestrial Mucorales (Mucoromycotina)
Source: PLoS Genet. 2014 Aug 14;10(8):e1004496. doi: 10.1371/journal.pgen.1004496 (PMC4133162; doi:10.1371/journal.pgen.1004496)
Supplement: Table S2 — Transposable and repetitive elements in the L. corymbifera genome. (PDF) [file pgen.1004496.s009.pdf]

| Transposable element | Total bases | Percent of assembly | Major superfamilies (% of class) |
| --- | --- | --- | --- |
| **DNA transposons** | 512,554 | 1.52 | |
| | | | EnSpan (23) |
| | | | Harbinger (7) |
| | | | hAT (14.7) |
| | | | Helitron (4.3) |
| | | | Mariner/Tc1 (5.3) |
| | | | MuDR (10.6) |
| | | | Polinton (9.8) |
| | | | Sola (4.8) |
| **LTR retrotransposons** | 612,555 | 1.82 | |
| | | | Gypsie (59.7) |
| | | | Copia (25.8) |
| | | | DIRS (6.4) |
| **Non- LTR retrotransposons** | 359,496 | 1.06 | |
| | | | Jockey (17.8) |
| | | | L1 (35.9) |
| | | | CR1 (14.6) |
| | | | R1 (7.4) |
| **Viruses*** | 65,316 | 0.19 | |

| | | |
|---|---|---|
| **Satellite and microsatellite** | 10,469 | 0.03 |
| **Simple repeats** | 18,155 | 0.05 |
| **Other repeats** | 17,634 | 0.05 |
| **Total** | 1,596,179 | 4.74 |

*DNA, integrated, endogeneous retrovirus
