# Supplementary material for: Gene Expansion Shapes Genome Architecture in the Human Pathogen Lichtheimia corymbifera: An Evolutionary Genomics Analysis in the Ancient Terrestrial Mucorales (Mucoromycotina)
Source: PLoS Genet. 2014 Aug 14;10(8):e1004496. doi: 10.1371/journal.pgen.1004496 (PMC4133162; doi:10.1371/journal.pgen.1004496)
Supplement: Table S3 — Overview of ncRNAs found in basal fungi. op – Close/Part to Operon; pg – Pseudogene; Ror – R. oryzae; Lco – L. corymbifera; ? – candidate. (PDF) [file pgen.1004496.s010.pdf]

ncRNA	Function	*Ror*	*Lco*	Remarks
5S rRNA	component of the large ribosomal subunit	2op	0op+1	
18S rRNA	small subunit of ribosomal RNA	2op	0	Within the assembly of *L. corymbifera*, several 100 nt fragments were found.
5.8S rRNA	component of the large ribosomal subunit	2op+1	1-4	In *L. corymbifera* found in read data only. Last hairpin divergent.
28S rRNA	component of the large ribosomal subunit	2op	0.5	In *L. corymbifera*, only 3' half was detected.
tRNA	transfer RNA	237+1pg	164+10pg	
Rnase P	tRNA processing	2	1	Central Pseudoknot of *L. corymbifera* diverse.
RNase MRP	rRNA processing	1	1	
SRP	directs ribosome to endoplasmatic reticulum	2	1	closer to metazoan than fungal SRP RNA
U1	major spliceosomal RNA	3	2	
U2	major spliceosomal RNA	4	2	
U4	major spliceosomal RNA	1-3	0-1	*L. corymbifera* gene was found in read data only.
U5	major spliceosomal RNA	3	2	

U6	major spliceosomal RNA	4	4	
U11	major spliceosomal RNA	1	1	*P. blakesleeanus* contains a 138 nt intron. Last stem longer.
U12	major spliceosomal RNA	1	1	Third stem atrophied. Last stem much shorter.
U4atac	minor spliceosomal RNA	1	0	
U6atac	minor spliceosomal RNA	1	1	Second half divergent.
U3	C/D box snoRNA, cleaves rRNA	4	3	
TPP	riboswitch, binds to thiamine pyrophosphate	4?	1?	
Telomerase	telomerase RNA component	0	1?	closer to ciliate than fungal telomerase RNA; No known proteins found
U7	histone processing RNA	-	4?	
