# Supplementary material for: Gene Expansion Shapes Genome Architecture in the Human Pathogen Lichtheimia corymbifera: An Evolutionary Genomics Analysis in the Ancient Terrestrial Mucorales (Mucoromycotina)
Source: PLoS Genet. 2014 Aug 14;10(8):e1004496. doi: 10.1371/journal.pgen.1004496 (PMC4133162; doi:10.1371/journal.pgen.1004496)
Supplement: Table S5 — Signalling pathway components included in the study and orthologues identified in L. corymbifera. (PDF) [file pgen.1004496.s012.pdf]

Name of orthologs	Gene ID	Assigned function
**SLN1**	LCor00162.1.t1<br>LCor00877.1.t1<br>LCor02767.1.t1<br>LCor03837.1.t1<br>LCor04582.1.t1<br>LCor05933.1.t1<br>LCor07413.1.t1<br>LCor07770.1.t1<br>LCor08728.1.t1<br>LCor09535.1.t1<br>LCor11074.1.t1<br>LCor11167.1.t1<br>LCor11494.1.t1<br>LCor11714.1.t1<br>LCor12256.1.t1*<br>LCor12257.1.t1*<br>LCor12312.1.t1	**Histidine kinase osmosensor that regulates a MAP kinase cascade; transmembrane protein with an intracellular kinase domain that signals to Ypd1p and Ssk1p, thereby forming a phosphorelay system similar to bacterial two component regulators**
**SLT2**	LCor06339.1.t1<br>LCor07992.1.t2	**Serine/threonine MAP kinase; involved in regulating maintenance of cell wall integrity, progression through the cell cycle, and nuclear mRNA retention in heat shock; required for mitophagy and pexophagy; affects recruitment of mitochondria to the phagophore assembly site (PAS); regulated by the PKC1 mediated signaling pathway**
**VCX1**	LCor01072.1.t1<br>LCor01703.1.t1<br>LCor02873.1.t1*<br>LCor02874.1.t1*<br>LCor03175.1.t1<br>LCor04086.1.t1<br>LCor07712.1.t1<br>LCor08010.1.t1<br>LCor10259.1.t1	**Vacuolar membrane antiporter with Ca2+/H+ and K+/H+ exchange activity, involved in control of cytosolic Ca2+ and K+ concentrations; has similarity to sodium/calcium exchangers, including the bovine Na+/Ca2+, K+ antiporter**
**YPS1**	LCor00186.1.t1<br>LCor02028.1.t1<br>LCor02157.1.t1*<br>LCor02158.1.t1*<br>LCor02462.1.t1*<br>LCor02463.1.t1*<br>LCor03453.1.t1<br>LCor03813.1.t1<br>LCor03873.1.t1<br>LCor05667.1.t1<br>LCor08268.1.t1<br>LCor08530.1.t1<br>LCor08737.1.t1<br>LCor09727.1.t1	**Aspartic protease, member of the yapsin family of proteases involved in cell wall growth and maintenance; attached to the plasma membrane via a glycosylphosphatidylinositol (GPI) anchor**

**BCK1**	LCor00818.1.t1<br>LCor08890.1.t1	**Mitogen activated protein (MAP) kinase kinase kinase acting in the protein kinase C signaling pathway, which controls cell integrity; upon activation by Pkc1p phosphorylates downstream kinases Mkk1p and Mkk2p**
**BCK2**	--	**Protein rich in serine and threonine residues involved in protein kinase C signaling pathway, which controls cell integrity; overproduction suppresses pkc1 mutations**
**BEM1**	LCor03278.1.t1<br>LCor04465.1.t1	**Protein containing SH3 domains, involved in establishing cell polarity and morphogenesis; functions as a scaffold protein for complexes that include Cdc24p, Ste5p, Ste20p, and Rsr1p**
**BEM2**	LCor00141.1.t1<br>LCor00193.1.t1<br>LCor01150.1.t1<br>LCor02720.1.t1<br>LCor05601.1.t1<br>LCor05860.1.t1<br>LCor07378.1.t1<br>LCor07454.1.t1<br>LCor07731.1.t1<br>LCor08677.1.t1<br>LCor09304.1.t1<br>LCor11327.1.t1<br>LCor11602.1.t1	**Rho GTPase activating protein (RhoGAP) involved in the control of cytoskeleton organization and cellular morphogenesis; required for bud emergence**
**BNI1**	LCor01281.1.t1<br>LCor01609.1.t1<br>LCor03044.1.t1<br>LCor04838.1.t1<br>LCor07542.1.t1<br>LCor09714.1.t1<br>LCor12294.1.t1	**Formin, nucleates the formation of linear actin filaments, involved in cell processes such as budding and mitotic spindle orientation which require the formation of polarized actin cables, functionally redundant with BNR1**
**CCH1**	LCor01676.1.t1<br>LCor07804.1.t1<br>LCor08750.1.t1<br>LCor10564.1.t1	**Voltage gated high affinity calcium channel involved in calcium influx in response to some environmental stresses as well as exposure to mating pheromones; interacts and colocalizes with Mid1p, suggesting Cch1p and Mid1p function together**
**CDC24**	LCor01389.1.t1<br>LCor01904.1.t1<br>LCor02636.1.t1<br>LCor06659.1.t1<br>LCor08112.1.t1<br>LCor11357.1.t1	**Guanine nucleotide exchange factor (GEF or GDP release factor) for Cdc42p; required for polarity establishment and maintenance, and mutants have morphological defects in bud formation and shmooing**
**CDC42**	LCor01683.1.t1<br>LCor07636.1.t1	**Small rho like GTPase, essential for establishment and maintenance of cell polarity; mutants have defects in the organization of actin and septins**

**CLA4**	LCor01190.1.t1<br>LCor02719.1.t1<br>LCor06587.1.t1	Cdc42pactivated signal transducing kinase of the PAK (p21activated kinase) family, along with Ste20p and Skm1p; involved in septin ring assembly, vacuole inheritance, cytokinesis, sterol uptake regulation; phosphorylates Cdc3p and Cdc10p
**CMD1**	LCor04968.1.t1<br>LCor06171.1.t1<br>LCor10629.1.t1	Calmodulin; Ca++ binding protein that regulates Ca++ independent processes (mitosis, bud growth, actin organization, endocytosis, etc.) and Ca++ dependent processes (stress activated pathways), targets include Nuf1p, Myo2p and calcineurin
**CMP2**	LCor02879.1.t2<br>LCor09841.1.t1	Calcineurin A; one isoform (the other is CNA1) of the catalytic subunit of calcineurin, a Ca++/calmodulin regulated protein phosphatase which regulates Crz1p (a stress response transcription factor), the other calcineurin subunit is CNB1
**CNA1**	LCor09841.1.t1<br>LCor02879.1.t2	Calcineurin A; one isoform (the other is CMP2) of the catalytic subunit of calcineurin, a Ca++/calmodulin regulated protein phosphatase which regulates Crz1p (a stress response transcription factor), the other calcineurin subunit is CNB1
**CNB1**	LCor05632.1.t1	Calcineurin B; the regulatory subunit of calcineurin, a Ca++/calmodulin regulated type 2B protein phosphatase which regulates Crz1p (a stress response transcription factor), the other calcineurin subunit is encoded by CNA1 and/or CMP1
**CPR1**	LCor00421.1.t1<br>LCor01642.1.t1<br>LCor02166.1.t1<br>LCor03788.1.t1<br>LCor05112.1.t1<br>LCor05866.1.t1<br>LCor07472.1.t1<br>LCor08282.1.t1<br>LCor09667.1.t1	Cytoplasmic peptidylprolyl cis-trans isomerase (cyclophilin), catalyzes the cis-trans isomerization of peptide bonds N' terminal to proline residues; binds the drug cyclosporin A
**CRZ1**	LCor00138.1.t1<br>LCor00424.1.t1<br>LCor01236.1.t1<br>LCor01802.1.t1<br>LCor01959.1.t1<br>LCor05575.1.t1<br>LCor12103.1.t1<br>LCor12188.1.t1	Transcription factor that activates transcription of genes involved in stress response; nuclear localization is positively regulated by calcineurin mediated dephosphorylation

**DIG1**	--	**MAP kinas responsive inhibitor of the Ste12p transcription factor, involved in the regulation of mating specific genes and the invasive growth pathway; related regulators Dig1p and Dig2p bind to Ste12p**
**DIG2**	--	**MAP kinas responsive inhibitor of the Ste12p transcription factor, involved in the regulation of mating specific genes and the invasive growth pathway; related regulators Dig1p and Dig2p bind to Ste12p**
**FAR1**	--	**Cyclin dependent kinase inhibitor that mediates cell cycle arrest in response to pheromone; also forms a complex with Cdc24p, Ste4p, and Ste18p that may specify the direction of polarized growth during mating; potential Cdc28p substrate**
**FIG1**	--	**Integral membrane protein required for efficient mating; may participate in or regulate the low affinity Ca2+ influx system, which affects intracellular signaling and cell cell fusion during mating**
**FPR1**	LCor02490.1.t1<br>LCor02934.1.t1<br>LCor05180.1.t1<br>LCor07780.1.t1<br>LCor08903.1.t1	**Peptidylprolyl cis-trans isomerase (PPIase), binds to the drugs FK506 and rapamycin; also binds to the non histone chromatin binding protein Hmo1p and may regulate its assembly or function**
**FUS1**	--	**Membrane protein localized to the shmoo tip, required for cell fusion; expression regulated by mating pheromone; proposed to coordinate signaling, fusion, and polarization events required for fusion; potential Cdc28p substrate**
**FUS3**	LCor04911.1.t1	**Mitogen activated serine/threonine protein kinase involved in mating; phospho-activated by Ste7p; substrates include Ste12p, Far1p, Bni1p, Sst2p; inhibits invasive growth during mating by phosphorylating Tec1p, promoting its degradation**
**GPA1**	LCor00522.1.t1<br>LCor02271.1.t1<br>LCor02939.1.t1<br>LCor02948.1.t1<br>LCor06277.1.t1<br>LCor06418.1.t1<br>LCor07162.1.t1<br>LCor07353.1.t1<br>LCor08481.1.t1<br>LCor10460.1.t1	**GTP binding alpha subunit of the heterotrimeric G protein that couples to pheromone receptors; negatively regulates the mating pathway by sequestering G (beta) gamma and by triggering an adaptive response; activates Vps34p at the endosome**

**HOG1**	LCor01571.1.t1<br>LCor01831.1.t1	**Mitogen activated protein kinase involved in osmoregulation; acts via three independent osmosensors; mitophagy specific regulator; mediates the recruitment and activation of RNA Pol II at Hot1pdependent promoters; localization regulated by Ptp2p and Ptp3p**
**HOT1**	LCor11021.1.t1	**Transcription factor required for the transient induction of glycerol biosynthetic genes GPD1 and GPP2 in response to high osmolarity; targets Hog1p to osmostress responsive promoters; has similarity to Msn1p and Gcr1p**
**HRR25**	LCor01591.1.t1<br>LCor09119.1.t3	**Protein kinase involved in regulating diverse events including vesicular trafficking, DNA repair, and chromosome segregation; binds the CTD of RNA pol II; homolog of mammalian casein kinase 1delta (CK1delta)**
**KSS1**	Same as FUS3	**Mitogen activated protein kinase (MAPK) involved in signal transduction pathways that control filamentous growth and pheromone response; the KSS1 gene is nonfunctional in S288C strains and functional in W303 strains**
**MCM1**	LCor01758.1.t1<br>LCor06393.1.t1<br>LCor07433.1.t1<br>LCor08527.1.t1<br>LCor08806.1.t1<br>LCor09108.1.t1<br>LCor10278.1.t1<br>LCor12313.1.t1	**Transcription factor involved in celltype specific transcription and pheromone response; plays a central role in the formation of both repressor and activator complexes**
**MF(ALPHA)1**	--	**Mating pheromone alpha factor, made by alpha cells; interacts with mating type a cells to induce cell cycle arrest and other responses leading to mating; also encoded by MF (ALPHA) 2, although MF (ALPHA) 1 produces most alpha factor**
**MF(ALPHA)2**	--	**Mating pheromone alpha factor, made by alpha cells; interacts with mating type a cells to induce cell cycle arrest and other responses leading to mating; also encoded by MF (ALPHA) 1, which is more highly expressed than MF (ALPHA) 2**
**MFA1**	--	**Mating pheromone a factor, made by a cells; interacts with alpha cells to induce cell cycle arrest and other responses leading to mating; biogenesis involves C' terminal modification, N′ terminal proteolysis, and export; also encoded by MFA2**

**MFA2**	--	**Mating pheromone a factor, made by a cells; interacts with alpha cells to induce cell cycle arrest and other responses leading to mating; biogenesis involves C' terminal modification, N′ terminal proteolysis, and export; also encoded by MFA1**
**MID1**	LCor10558.1.t1	**N' glycosylated integral membrane protein of the ER membrane and plasma membrane, functions as a stretch activated Ca2+ permeable cation channel required for Ca2+ influx stimulated by pheromone; interacts with Cch1p**
**MID2**	--	**O glycosylated plasma membrane protein that acts as a sensor for cell wall integrity signaling and activates the pathway; interacts with Rom2p, a guanine nucleotide exchange factor for Rho1p, and with cell integrity pathway protein Zeo1p**
**MKK1**	LCor03536.1.t1<br>LCor04751.1.t1<br>LCor08249.1.t1	**Mitogen activated kinase kinase involved in protein kinase C signaling pathway that controls cell integrity; upon activation by Bck1p phosphorylates downstream target, Slt2p; functionally redundant with Mkk2p**
**MSB2**	--	**Mucin family member involved in the Cdc42p and MAP kinase dependent filamentous growth signaling pathway; also functions as an osmosensor in parallel to the Sho1p mediated pathway; potential Cdc28p substrate**
**MSG5**	LCor05433.1.t1	**Dual specificity protein phosphatase; exists in 2 isoforms; required for maintenance of a low level of signaling through the cell integrity pathway, adaptive response to pheromone; regulates and is regulated by Slt2p; dephosphorylates Fus3p**
**MSN1**	--	**Transcriptional activator involved in regulation of invertase and glucoamylase expression, invasive growth and pseudohyphal differentiation, iron uptake, chromium accumulation, and response to osmotic stress; localizes to the nucleus**
**MSN2**	--	**Transcriptional activator related to Msn4p; activated in stress conditions, which results in translocation from the cytoplasm to the nucleus; binds DNA at stress response elements of responsive genes, inducing gene expression**

**MSN4**	LCor00249.1.t1<br>LCor01478.1.t1<br>LCor02196.1.t1<br>LCor04997.1.t1<br>LCor07383.1.t1	**Transcriptional activator related to Msn2p; activated in stress conditions, which results in translocation from the cytoplasm to the nucleus; binds DNA at stress response elements of responsive genes, inducing gene expression**
**MTL1**	--	**Putative plasma membrane sensor, involved in cell integrity signaling and stress response during glucose starvation and oxidative stress; has structural and functional similarity to Mid2p**
**PBS2**	LCor00564.1.t1	**MAP kinase kinase of the HOG signaling pathway; activated under severe osmotic stress; mitophagy specific regulator; plays a role in regulating Ty1 transposition**
**PIR3**	--	**O glycosylated covalently bound cell wall protein required for cell wall stability; expression is cell cycle regulated, peaking in M/G1 and also subject to regulation by the cell integrity pathway**
**PKC1**	LCor02728.1.t1<br>LCor04156.1.t1	**Protein serine/threonine kinase essential for cell wall remodeling during growth; localized to sites of polarized growth and the mother daughter bud neck; homolog of the alpha, beta, and gamma isoforms of mammalian protein kinase C (PKC)**
**PMC1**	LCor02317.1.t1<br>LCor07996.1.t1<br>LCor06558.1.t1<br>LCor10117.1.t1<br>LCor07729.1.t1<br>LCor11865.1.t1	**Vacuolar Ca2+ ATPase involved in depleting cytosol of Ca2+ ions; prevents growth inhibition by activation of calcineurin in the presence of elevated concentrations of calcium; similar to mammalian PMCA1a**
**PMR1**	LCor11533.1.t1	**High affinity Ca2+/Mn2+ Ptype ATPase required for Ca2+ and Mn2+ transport into Golgi; involved in Ca2+ dependent protein sorting and processing; mutations in human homolog ATP2C1 cause a cantholytic skin condition Hailey Hailey disease**
**PPZ1/PPZ2**	LCor01164.1.t1<br>LCor03163.1.t1<br>LCor03495.1.t1	**Serine/threonine protein phosphatases Z, isoform of Ppz2p; involved in regulation of potassium transport, which affects osmotic stability, cell cycle progression, and halo tolerance**
**PRM5**	--	**Pheromone regulated protein, predicted to have 1 transmembrane segment; induced during cell integrity signaling**

**PST1**	LCor04890.1.t1<br>LCor12329.1.t1<br>LCor04695.1.t1<br>LCor06520.1.t1	**Cell wall protein that contains a putative GPI attachment site; secreted by regenerating protoplasts; up regulated by activation of the cell integrity pathway, as mediated by Rlm1p; up regulated by cell wall damage via disruption of FKS1**
**RAS2**	LCor00430.1.t1<br>LCor03725.1.t1<br>LCor04610.1.t1<br>LCor06688.1.t1	**GTP binding protein that regulates the nitrogen starvation response, sporulation, and filamentous growth; farnesylation and palmitoylation required for activity and localization to plasma membrane; homolog of mammalian Ras proto oncogenes**
**RCK1/RCK2**	LCor00478.1.t1<br>LCor03419.1.t1<br>LCor03168.1.t1<br>LCor06432.1.t1<br>LCor07694.1.t1	**Protein kinases involved in the response to oxidative stress; identified as suppressor of S. pombe cell cycle checkpoint mutations**
**RCN1**	--	**Protein involved in calcineurin regulation during calcium signaling; has similarity to H. sapiens DSCR1 which is found in the Down Syndrome candidate region**
**RHO1**	LCor08052.1.t2	**GTP binding protein of the rho subfamily of Ras like proteins, involved in establishment of cell polarity; regulates protein kinase C (Pkc1p) and the cell wall synthesizing enzyme 1,3 beta-glucan synthase (Fks1p and Gsc2p)**
**RHO5**	--	**Non essential small GTPase of the Rho/Rac subfamily of Ras like proteins, likely involved in protein kinase C (Pkc1p) dependent signal transduction pathway that controls cell integrity**
**RLM1**	LCor05504.1.t1	**MADS box transcription factor, component of the protein kinase C mediated MAP kinase pathway involved in the maintenance of cell integrity; phosphorylated and activated by the MAP kinase Slt2p**
**ROM1**	LCor01841.1.t1<br>LCor02021.1.t1<br>LCor05488.1.t2<br>LCor07499.1.t1<br>LCor10007.1.t1	**GDP/GTP exchange proteins (GEP) for Rho1p; mutations are synthetically lethal with mutations in rom2, which also encodes a GEP**
**RTN1**	--	**ER membrane protein that interacts with Sey1p to maintain ER morphology; interacts with exocyst subunit Sec6p, with Yip3p, and with Sbh1p; null mutant has an altered ER morphology; member of the RTNLA (reticulon like A) subfamily**

**RTN2**	--	**Protein of unknown function; has similarity to mammalian reticulon proteins; member of the RTNLA (reticulon like A) subfamily**
**SAC7**	LCor02140.1.t1<br>LCor06413.1.t1<br>LCor10643.1.t1	**GTPase activating protein (GAP) for Rho1p, involved in signaling to the actin cytoskeleton, null mutations suppress tor2 mutations and temperature sensitive mutations in actin; potential Cdc28p substrate**
**SHO1**	LCor00503.1.t1<br>LCor01615.1.t1<br>LCor04563.1.t1<br>LCor07332.1.t1<br>LCor07854.1.t1<br>LCor08971.1.t1	**Transmembrane osmosensor involved in activation of the Cdc42p and MAP kinase dependent filamentous growth pathway and the high osmolarity glycerol response pathway; phosphorylated by Hog1p; interacts with Pbs2p, Msb2p, Hkr1p, and Ste11p**
**SIT4**	LCor02322.1.t1<br>LCor03480.1.t1<br>LCor03616.1.t1<br>LCor07635.1.t1<br>LCor09474.1.t1<br>LCor10112.1.t1	**Type 2A related serine/threonine phosphatase that functions in the G1/S transition of the mitotic cycle; cytoplasmic and nuclear protein that modulates functions mediated by Pkc1p including cell wall and actin cytoskeleton organization**
**SKO1**	--	**Basic leucine zipper transcription factor of the ATF/CREB family; forms a complex with Tup1p and Cyc8p to both activate and repress transcription; cytosolic and nuclear protein involved in osmotic and oxidative stress responses**
**SLG1**	--	**Sensor transducer of the stress activated PKC1 MPK1 kinase pathway; involved in maintenance of cell wall integrity; required for mitophagy; involved in organization of the actin cytoskeleton; secretory pathway Wsc1p is required for the arrest of secretion response**
**SMP1**	LCor03918.1.t1<br>LCor08105.1.t1<br>LCor08386.1.t1	**Putative transcription factor involved in regulating the response to osmotic stress; member of the MADS box family of transcription factors**
**SSK1**	LCor05616.1.t1<br>LCor09508.1.t1	**Cytoplasmic response regulator; part of a two component signal transducer that mediates osmo-sensing via a phosphorelay mechanism; required for mitophagy; dephosphorylated form is degraded by the ubiquitin proteasome system; potential Cdc28p substrate**

**SSK2**	--	**MAP kinase kinase kinase of the HOG1 mitogen activated signaling pathway; interacts with Ssk1p, leading to autophosphorylation and activation of Ssk2p which phosphorylates Pbs2p; also mediates actin cytoskeleton recovery from osmotic stress**
**SSK22**	LCor02371.1.t1<br>LCor09478.1.t1	**MAP kinase kinase kinase of the HOG1 mitogen activated signaling pathway; functionally redundant with, and homologous to, Ssk2p; interacts with and is activated by Ssk1p; phosphorylates Pbs2p**
**STE11**	LCor09357.1.t1	**Signal transducing MEK kinase involved in pheromone response and pseudohyphal / invasive growth pathways where it phosphorylates Ste7p, and the high osmolarity response pathway, via phosphorylation of Pbs2p; regulated by Ste20p and Ste50p**
**STE12**	LCor01878.1.t2<br>LCor11169.1.t1	**Transcription factor that is activated by a MAP kinase signaling cascade, activates genes involved in mating or pseudohyphal / invasive growth pathways; cooperates with Tec1p transcription factor to regulate genes specific for invasive growth**
**STE18**	LCor03271.1.t1	**G protein gamma subunit, forms a dimer with Ste4p to activate the mating signaling pathway, forms a heterotrimer with Gpa1p and Ste4p to dampen signaling; C' terminus is palmitoylated and farnesylated, which are required for normal signaling**
**STE2**	--	**Receptor for alpha factor pheromone; seven transmembrane domain GPCR that interacts with both pheromone and a heterotrimeric G protein to initiate the signaling response that leads to mating between haploid a and alpha cells**
**STE20**	LCor01168.1.t1<br>LCor04274.1.t1<br>LCor05516.1.t1<br>LCor06936.1.t1<br>LCor07069.1.t1	**Cdc42p activated signal transducing kinase of the PAK (p21activated kinase) family; involved in pheromone response, pseudohyphal/invasive growth, vacuole inheritance, down regulation of sterol uptake; GBB motif binds Ste4p**
**STE3**	--	**Receptor for a factor pheromone, couples to MAP kinase cascade to mediate pheromone response; transcribed in alpha cells and required for mating by alpha cells, ligand bound receptors endocytosed and recycled to the plasma membrane; GPCR**

**STE4**	LCor01272.1.t1<br>LCor07294.1.t1<br>LCor07816.1.t1<br>LCor09889.1.t1<br>LCor10582.1.t1	**G protein beta subunit, forms a dimer with Ste18p to activate the mating signaling pathway, forms a heterotrimer with Gpa1p and Ste18p to dampen signaling; may recruit Rho1p to the polarized growth site during mating; contains WD40 repeats**
**STE5**	--	**Pheromone response scaffold protein that controls the mating decision; binds Ste11p, Ste7p, and Fus3p kinases, forming a MAPK cascade complex that interacts with the plasma membrane and Ste4p Ste18p; allosteric activator of Fus3p**
**STE50**	--	**Protein involved in mating response, invasive/filamentous growth, and osmo-tolerance, acts as an adaptor that links G protein associated Cdc42p Ste20p complex to the effector Ste11p to modulate signal transduction**
**STE7**	LCor05404.1.t1	**Signal transducing MAP kinase kinase involved in pheromone response, where it phosphorylates Fus3p, and in the pseudohyphal/invasive growth pathway, through phosphorylation of Kss1p; phosphorylated by Ste11p, degraded by ubiquitin pathway**
**SWI4**	LCor01791.1.t1<br>LCor07806.1.t1<br>LCor09533.1.t1	**DNA binding component of the SBF complex (Swi4p Swi6p), a transcriptional activator that in concert with MBF (Mbp1 Swi6p) regulates late G1specific transcription of targets including cyclins and genes required for DNA synthesis and repair**
**TEC1**	LCor03533.1.t1<br>LCor08455.1.t1	**Transcription factor required for full Ty1 expression, Ty1 mediated gene activation, and haploid invasive and diploid pseudohyphal growth; TEA/ATTS DNA binding domain family member**
**TUS1**	LCor01842.1.t1<br>LCor04938.1.t1<br>LCor05487.1.t1	**Guanine nucleotide exchange factor (GEF) that functions to modulate Rho1p activity as part of the cell integrity signaling pathway; multicopy suppressor of tor2 mutation and ypk1 ypk2 double mutation; potential Cdc28p substrate**
**WSC2**	--	**Partially redundant sensor transducer of the stress activated PKC1 MPK1 signaling pathway involved in maintenance of cell wall integrity and recovery from heat shock; secretory pathway Wsc2p is required for the arrest of secretion response**

**WSC3**	--	**Partially redundant sensor transducer of the stress activated PKC1 MPK1 signaling pathway involved in maintenance of cell wall integrity; involved in the response to heat shock and other stressors; regulates 1,3 beta glucan synthesis**
**YPD1**	LCor00246.1.t1<br>LCor01065.1.t1	**Phosphorelay intermediate protein, phosphorylated by the plasma membrane sensor Sln1p in response to osmotic stress and then in turn phosphorylates the response regulators Ssk1p in the cytosol and Skn7p in the nucleus**
**YVC1**	--	**Vacuolar cation channel, mediates release of Ca (2+) from the vacuole in response to hyper osmotic shock**
**ZEO1**	--	**Peripheral membrane protein of the plasma membrane that interacts with Mid2p; regulates the cell integrity pathway mediated by Pkc1p and Slt2p; the authentic protein is detected in a phosphorylated state in highly purified mitochondria**

Genes marked with "*" are probable duplications
