# Supplementary material for: Gene Expansion Shapes Genome Architecture in the Human Pathogen Lichtheimia corymbifera: An Evolutionary Genomics Analysis in the Ancient Terrestrial Mucorales (Mucoromycotina)
Source: PLoS Genet. 2014 Aug 14;10(8):e1004496. doi: 10.1371/journal.pgen.1004496 (PMC4133162; doi:10.1371/journal.pgen.1004496)
Supplement: Table S6 — Classification and gene IDs of putative protein phosphatases in the L. corymbifera genome. (PDF) [file pgen.1004496.s013.pdf]

	S. cerevisiae	*R. oryzae*	*L. corymbifera*
**CDC25**			
	SCRG_01933	none	none
**DSPs**			
	SCRG_05285	RO3G_01445	LCor00777.1.t1
	SCRG_05575	RO3G_00853	LCor02142.1.t1
	SCRG_05152	RO3G_00502	LCor04949.1.t1
	SCRG_02694	RO3G_07214	LCor05281.1.t4
	SCRG_03190	RO3G_03637	LCor05433.1.t1
	SCRG_03261	RO3G_12794	LCor05489.1.t1
		RO3G_15103	LCor06286.1.t1
		RO3G_16335	LCor08356.1.t1
		RO3G_04063	LCor08468.1.t1
		RO3G_08234	LCor09285.1.t1
			LCor09981.1.t1
			LCor10898.1.t1
			LCor10947.1.t1
			LCor11198.1.t1
**FCP1**			
	SCRG_02173	RO3G_04929	LCor02318.1.t1
		RO3G_16681	
**LmwPTP**			
	SCRG_02557	RO3G_16977	LCor09244.1.t1
**Myotubularin**			
	SCRG_03760	RO3G_06890	LCor05331.1.t1
**PTPs**			
	SCRG_00717	RO3G_00326	LCor05981.1.t1
	SCRG_04551	RO3G_04458	LCor09442.1.t1
	SCRG_01596	RO3G_06503	LCor06192.1.t1
	SCRG_03193	RO3G_07589	LCor08202.1.t1
	SCRG_03158	RO3G_08421	LCor09938.1.t1
	SCRG_00448	RO3G_11155	
	SCRG_03235	RO3G_02273	
		RO3G_14132	
		RO3G_13499	
		RO3G_13912	
**STPPs**			
	SCRG_04788	RO3G_01038	LCor00604.1.t1
	SCRG_00547	RO3G_02253	LCor03810.1.t2
	SCRG_04609	RO3G_07220	LCor04784.1.t2
	SCRG_01879	RO3G_13180	LCor00797.1.t1
	SCRG_02318	RO3G_14156	LCor01164.1.t1
	SCRG_01842	RO3G_15330	LCor01800.1.t1
	SCRG_04371	RO3G_16219	LCor02322.1.t1
	SCRG_03111	RO3G_02173	LCor02879.1.t2
	SCRG_00440	RO3G_04542	LCor03163.1.t1
	SCRG_00679	RO3G_04836	LCor03480.1.t1
	SCRG_00630	RO3G_00528	LCor03495.1.t1

	SCRG_00895	RO3G_12129	LCor03616.1.t2
	SCRG_00101	RO3G_02565	LCor06144.1.t1
	SCRG_03343	RO3G_08858	LCor06724.1.t1
		RO3G_10916	LCor07635.1.t1
		RO3G_14499	LCor08272.1.t1
		RO3G_13122	LCor09474.1.t1
		RO3G_13385	LCor09841.1.t1
		RO3G_12832	LCor10112.1.t1
		RO3G_14483	LCor10351.1.t1
		RO3G_08936	
		RO3G_00628	
		RO3G_04139	
		RO3G_12341	
		RO3G_13249	
		RO3G_13891	
		RO3G_14955	
		RO3G_16278	
		RO3G_10591	
		RO3G_03043	
**PP2Cs**			
	SCRG_04566	RO3G_01402	LCor00327.1.t1
	SCRG_02843	RO3G_06856	LCor00363.1.t1
	SCRG_00513	RO3G_06853	LCor01216.1.t1
	SCRG_01488	RO3G_10282	LCor01254.1.t1
	SCRG_03018	RO3G_02940	LCor04110.1.t1
	SCRG_05468	RO3G_04097	LCor07086.1.t1
		RO3G_01541	LCor07733.1.t1
		RO3G_06955	LCor09007.1.t1
		RO3G_11555	LCor09282.1.t1
		RO3G_14392	LCor07825.1.t1
		RO3G_16439	
**Total**	**37**	**65**	**52**
