# Supplementary material for: Gene Expansion Shapes Genome Architecture in the Human Pathogen Lichtheimia corymbifera: An Evolutionary Genomics Analysis in the Ancient Terrestrial Mucorales (Mucoromycotina)
Source: PLoS Genet. 2014 Aug 14;10(8):e1004496. doi: 10.1371/journal.pgen.1004496 (PMC4133162; doi:10.1371/journal.pgen.1004496)
Supplement: Table S9 — Genomes used in this study. (PDF) [file pgen.1004496.s016.pdf]

Species name	Proteome	Source	Genome project
Aspergillus fumigatus	ASPFU.4	UniProt reference genomes	*Aspergillus* Comparative Sequencing Project, Broad Institute of Harvard and MIT (http://www.broadinstitute.org/)
Aspergillus nidulans	ANID.4	Broad Institute	*Aspergillus* Comparative Sequencing Project, Broad Institute of Harvard and MIT (http://www.broadinstitute.org/)
Batrachochytrium dendrobatidis	BATDE.2	Broad Institute	*Batrachochytrium dendrobatidis* Sequencing Project, Broad Institute of Harvard and MIT (http://www.broadinstitute.org/)
Cryptococcus neoformans	CRYNE.3	Broad Institute	*Cryptococcus neoformans var. grubii* H99 Sequencing Project, Broad Institute of Harvard and MIT (http://www.broadinstitute.org/)
Encephalitozoon cuniculi	ENCCU.4	Broad Institute	Microsporidia Comparative Sequencing Project, Broad Institute of Harvard and MIT (http://www.broadinstitute.org/)
Homoloaphlyctis polyrhiza	HOMPO.1	GenBank PRJNA68115	*Homoloaphlyctis polyrhiza* JEL 142 genome project
Laccaria bicolor	LACBI.2	U.S. Department of Energy Joint Genome Institute (DOE JGI)	*Laccaria bicolor* genome project. U.S. Department of Energy Joint Genome Institute (DOE JGI) managed by Lawrence Berkeley National Laboratory (http://genome.jgi-psf.org/Lacbi2/Lacbi2.home.html)
Monosiga brevicollis	MONBE.2	U.S. Department of Energy Joint Genome Institute (DOE JGI)	*Monosiga brevicollis* genome project. U.S. Department of Energy Joint Genome Institute (DOE JGI) managed by Lawrence Berkeley National Laboratory (http://genome.jgi-psf.org/Monbr1/Monbr1.home.html)
Mortierella alpina	685557,1	GenBank PRJNA41211	*Mortierella alpina* genome project
Mucor circinelloides	MUCCI.1	U.S. Department of Energy Joint Genome Institute (DOE JGI)	*Mucor circinelloides* genome project. U.S. Department of Energy Joint Genome Institute (DOE JGI) managed by Lawrence Berkeley National Laboratory (http://genome.jgi-psf.org/Mucci2/Mucci2.home.html)
Nematocida parisii	586133,1	Broad Institute	Microsporidia Comparative Sequencing Project, Broad Institute of Harvard and MIT (http://www.broadinstitute.org/)
Nematostella vectensis	NEMVE.1	U.S. Department of Energy Joint Genome Institute (DOE JGI)	*Nematostella vectensis* genome project. U.S. Department of Energy Joint Genome Institute (DOE JGI) managed by Lawrence Berkeley National Laboratory (http://genome.jgi-psf.org/Nemve1/Nemve1.home.html)
Neurospora crassa	NEUCR.5	UniProt reference genomes	*Neurospora crassa*Sequencing Project, Broad Institute of Harvard and MIT (http://www.broadinstitute.org/)
Nosema ceranae (strain BRL01)	NOSCE.2	Broad Institute	Microsporidia Comparative Sequencing Project, Broad Institute of Harvard and MIT (http://www.broadinstitute.org/)
Paracoccidioides brasiliensis	PARBR.2	Broad Institute	*Paracoccidioides brasiliensis* Sequencing Project, Broad Institute of Harvard and MIT (http://www.broadinstitute.org/)
Phanerochaete chrysosporium	PHACH.1	U.S. Department of Energy Joint Genome Institute (DOE JGI)	*Phanerochaete chrysosporium* genome project. U.S. Department of Energy Joint Genome Institute (DOE JGI) managed by Lawrence Berkeley National Laboratory (http://genome.jgi-psf.org/Phchr1/Phchr1.home.html)

Phycomyces blakesleeanus	PHYBL.2	U.S. Department of Energy Joint Genome Institute (DOE JGI)	*Phycomyces blakesleeanus* genome project. U.S. Department of Energy Joint Genome Institute (DOE JGI) managed by Lawrence Berkeley National Laboratory (http://genome.jgi-psf.org/Phybl2/Phybl2.home.html)
Puccinia graminis	PUCGR.2	Broad Institute	*Puccinia* Group Sequencing Project, Broad Institute of Harvard and MIT (http://www.broadinstitute.org/)
Rhizopus oryzae	RHIOR.2	Broad Institute	*Rhizopus oryzae* Sequencing Project, Broad Institute of Harvard and MIT (http://www.broadinstitute.org/)
Saccharomyces cerevisiae	YEAST.5	Saccharomyces Genome Database (SGD)	*Saccharomyces cerevisiae* genome project
Schizosaccharomyces pombe (strain 972/ATCC 24843)	SCHPO.3	Broad Institute	*Schizosaccharomyces* Group Sequencing Project, Broad Institute of Harvard and MIT (http://www.broadinstitute.org/)
Serpula lacrymans var. *lacrymans* S7.9	SERL9.1	U.S. Department of Energy Joint Genome Institute (DOE JGI)	*Serpula lacrymans* genome project. U.S. Department of Energy Joint Genome Institute (DOE JGI) managed by Lawrence Berkeley National Laboratory (http://genome.jgi-psf.org/SerlaS7_9_2/SerlaS7_9_2.home.html)
Ustilago maydis	USTMA.4	Quest For Orthologs:RELEASE 2011_04	*Ustilago maydis* Sequencing Project. Broad Institute of MIT and Harvard (http://www.broadinstitute.org/)
